# The antiproliferative ELF2 isoform, ELF2B, induces apoptosis in vitro and perturbs early lymphocytic development in vivo

**DOI:** 10.1186/s13045-017-0446-7

**Published:** 2017-03-28

**Authors:** Fiona H. X. Guan, Charles G. Bailey, Cynthia Metierre, Patrick O’Young, Dadi Gao, Teh Liane Khoo, Jeff Holst, John E. J. Rasko

**Affiliations:** 1 Gene and Stem Cell Therapy Program, Centenary Institute, University of Sydney, Camperdown, NSW 2050 Australia; 2 Sydney Medical School, University of Sydney, Camperdown, NSW 2006 Australia; 3 Origins of Cancer Program, Centenary Institute, University of Sydney, Camperdown, NSW 2050 Australia; 4 Cell and Molecular Therapies, Royal Prince Alfred Hospital, Camperdown, NSW 2050 Australia

**Keywords:** ELF2, ELF2A, ELF2B, Isoform, DNA binding, Ets domain, Transcription factor, Dominant negative, Antiproliferative, Apoptosis, Lymphoid development, Tumour suppressor

## Abstract

**Background:**

ELF2 (E74-like factor 2) also known as NERF (new *Ets*-related factor), a member of the Ets family of transcription factors, regulates genes important in B and T cell development, cell cycle progression, and angiogenesis. Conserved ELF2 isoforms, ELF2A, and ELF2B, arising from alternative promoter usage can exert opposing effects on target gene expression. ELF2A activates, whilst ELF2B represses, gene expression, and the balance of expression between these isoforms may be important in maintaining normal cellular function.

**Methods:**

We compared the function of ELF2 isoforms ELF2A and ELF2B with other ELF subfamily proteins ELF1 and ELF4 in primary and cancer cell lines using proliferation, colony-forming, cell cycle, and apoptosis assays. We further examined the role of ELF2 isoforms in haemopoietic development using a *Rag1* ^-/-^murine bone marrow reconstitution model.

**Results:**

ELF2B overexpression significantly reduced cell proliferation and clonogenic capacity, minimally disrupted cell cycle kinetics, and induced apoptosis. In contrast, ELF2A overexpression only marginally reduced clonogenic capacity with little effect on proliferation, cell cycle progression, or apoptosis. Deletion of the N-terminal 19 amino acids unique to ELF2B abrogated the antiproliferative and proapoptotic functions of ELF2B thereby confirming its crucial role. Mice expressing Elf2a or Elf2b in haemopoietic cells variously displayed perturbations in the pre-B cell stage and multiple stages of T cell development. Mature B cells, T cells, and myeloid cells in steady state were unaffected, suggesting that the main role of ELF2 is restricted to the early development of B and T cells and that compensatory mechanisms exist. No differences in B and T cell development were observed between ELF2 isoforms.

**Conclusions:**

We conclude that ELF2 isoforms are important regulators of cellular proliferation, cell cycle progression, and apoptosis. In respect to this, ELF2B acts in a dominant negative fashion compared to ELF2A and as a putative tumour suppressor gene. Given that these cellular processes are critical during haemopoiesis, we propose that the regulatory interplay between ELF2 isoforms contributes substantially to early B and T cell development.

**Electronic supplementary material:**

The online version of this article (doi:10.1186/s13045-017-0446-7) contains supplementary material, which is available to authorized users.

## Background

Unprecedented insights into the global interaction of transcription factors with DNA, often in a tissue-specific context, have become available consequent to next generation sequencing technologies. It is necessary to understand the complex interplay between DNA sequence, protein structure, and protein-protein interactions (PPIs) in determining gene regulatory pathways. The *Ets* (E-twenty-six) family of transcription factors, characterised by the presence of an evolutionarily conserved 85 amino acid (aa) *Ets* DNA-binding domain, utilises a range of factors to govern target specificity. *Ets* proteins are classified into subfamilies based on sequence similarity in the *Ets* domain and by flanking domains, which can determine whether they act positively or negatively as transcriptional regulators. In humans, 27 members of the *Ets* family have been characterised, and many function as critical mediators of a wide variety of cellular processes, which include embryonic development, differentiation, growth, apoptosis, and oncogenic transformation [1–3].

The *Ets* domain forms a winged helix-turn-helix structure that binds the core *Ets* motif 5′-GGAA/T-3′ [4, 5]. Outside of the core sequence, the *Ets* domain has high tolerance of variations in its target sequence [6]. A key question is how *Ets* proteins orchestrate DNA binding specificity to regulate specific biological processes. Analysis of individual *Ets* family member DNA binding sites has indicated that specific as well as redundant occupancy may occur at *Ets* sites throughout the genome [7]. Subtle differences in *Ets* sites, tissue-specific expression of *Ets* factors and their co-factors, and differential signalling responses may all contribute to their distinct functions, but makes identifying true targets both problematic and challenging [8, 9].

Certain *Ets* proteins are known to play important roles in haemopoietic development via transcriptional regulation. Knockout mouse models have helped unravel the functional importance of *Ets* proteins in haemopoiesis. Loss of PU.1 (SPI1) has a profound effect on haemopoietic development by affecting myeloid and B cell development [10, 11]. Other *Ets* gene knockout mouse models with defects in haemopoietic cells include *Ets1* [12, 13], *SpiB* [14], *Fli1* [15], and *Etv6* [16]. Members of the ELF (E74-like factor) subfamily of Ets transcription factors including ELF1, ELF2, and ELF4 also play important roles in the development of lymphocytes and regulate numerous haemopoietic-specific genes. ELF1, which regulates genes involved in T cell development such as CD4 [17], CD3ζ [18], and IL-2 [19], also plays a restricted role in natural killer T cell development [20]. ELF4 (MEF; myeloid ELF-1-like factor) distinctly plays a critical role in the development and function of natural killer cells [21]. ELF2, also known as NERF (new Ets-related factor), is the least characterised member of this subfamily, despite its identification by two independent groups over 20 years ago [22, 23]. ELF2 binds to the regulatory regions of genes involved in lymphocyte development and function including B and T cell co-receptor proteins, tyrosine kinases, and enhancer regions [23–25]; and in many instances, is shown to modulate their expression levels. A knockout mouse model for ELF2 has not been reported, so little is known about its functional role in haemopoietic development.

Two major isoforms of ELF2 arise from alternative promoter usage, ELF2A (NERF-2), and ELF2B (NERF-1) [23]. These major isoforms of ELF2 can exhibit opposite regulatory effects, ELF2A activates whilst ELF2B represses expression of its target genes [24]. Importantly, both isoforms interact with the master haemopoietic regulators RUNX1 and LMO2 [22, 24]. Whilst both isoforms can bind the same *Ets* target sites in DNA and bind common co-factors, little is known about what functional differences these ELF2 isoforms may have.

In this report, we established reagents to distinguish between ELF2 isoforms and showed that ELF2 isoforms are differentially expressed. Our overexpression studies comparing between the ELF2 isoforms and the related ELF family members ELF1 and ELF4 in primary and transformed cell lines demonstrated a proapoptotic role for ELF2B which was modulated through its N-terminus. We then explored the role of ELF2 isoforms in haemopoietic development using an in vivo bone marrow reconstitution model in *Rag1* ^-/-^ mice. Our results show a defined effect on B and T cells as well as granulocytes, consistent with a potential role for ELF2 in regulating haemopoietic development.

## Methods

### Vector construction

Full-length human ELF1 and ELF4 cDNAs were obtained from cDNA prepared from human thymus total RNA whilst ELF2A and ELF2B cDNAs were obtained from cDNA prepared from human testis total RNA (FirstChoice® Human Total RNA Survey Panel, Ambion). Mouse Elf2 isoforms were amplified from cDNA prepared from mouse testis RNA. Each full-length cDNA sequence was then cloned into the pcDNA3.1-HA expression vector containing a haemagglutinin (HA) tag on the N-terminus using *Not*I and *Xba*I sites. ELF2Δ, representing the common 513 aa region of ELF2 isoforms was amplified from ELF2A using primers with *NotI*-5’ and *ClaI*-3’ ends and was cloned into pcDNA3.1-HA. To construct lentiviral vectors, each HA-tagged ELF gene was subcloned into pCCLteteGFP-2A lentiviral vector [26] via *BmgBI* and *ClaI* sites. To construct retroviral vectors, each Elf2 isoform was subcloned into the pMIG retroviral vector upstream of the IRES sequence via *BamHI* and *PmeI* sites. Primer sequences used for cloning are available on request.

### Cell culture

HeLa, HEK293T, MPRO, and GP + E86 ecotropic retrovirus packaging cells were cultured in DMEM (MPRO with 10% (v/v) conditioned DMEM medium from BHK-HM5 cells secreting GM-CSF). K562, Jurkat, A20 and CH12 cells were grown in RPM1 1640 medium (A20 and CH12 cells with the addition of 50 μM β-mercaptoethanol (Sigma-Aldrich). All basal media were supplemented with 10% FCS (v/v), penicillin (100 U/mL), and streptomycin (100 μg/mL). Human foreskin fibroblast (hFF) cells were grown in Ham’s F-12K (Kaighn’s) media supplemented with 50 μg/mL ascorbic acid (Sigma-Aldrich), 5 ng/mL basic fibroblast growth factor (PeproTech), 1 μg/mL hydrocortisone (Sigma-Aldrich), 5 μg/mL bovine insulin (Sigma-Aldrich), and 20% v/v FCS. All cell lines are routinely tested for *Mycoplasma* contamination by PCR screening of genomic DNA isolates.

### Lentivirus and retrovirus production

Lentiviral particles were produced using a four plasmid tat-independent packaging system delivered into cells by calcium phosphate transfection [27]. At approximately 16 h post-transfection, the medium was replaced with fresh DMEM supplemented with 5 mM sodium butyrate. The media was collected after 24 h, and the virus-containing media was filtered through a 0.45-μM filter (MillexHV Millipore) to remove cell debris. Viral concentration was achieved by centrifugation at 20,000*g* for 2 h at 4 °C in a Beckman L8-70M Ultracentrifuge using an SW28 rotor (Beckman). Following centrifugation, the supernatant was removed, and the viral pellets were resuspended in 1/100th of the original volume in DMEM/10% FCS. Viral titres were determined by testing transduction levels on HeLa cells using serially diluted virus. Cells were collected 48 h post-transduction and analysis by flow cytometry using an LSR Fortessa (BD). Percentages of GFP-positive cells at each virus dilution were evaluated using FlowJo version 9.4 (Treestar).

### Gene expression analysis

Total RNA was extracted from mouse tissues or immortalised cell lines using TRI Reagent (Astral Scientific). Each RNA sample was first treated with DNase I before generation of oligo dT cDNA by reverse transcription using SuperScript III (Invitrogen). After each RT reaction, the samples were treated with RNase H (New England BioLabs). Gene expression levels were quantified using the CFX96 Touch™ Real-Time PCR Detection System (BioRad) in 10 μL reactions, containing 25 ng of cDNA template, SYBR green-containing iQ Master Mix buffer (BioRad), 300 nM of forward and reverse primers (Additional file 1: Table S1), and UltraPure™ DNase/RNase-Free distilled water (Invitrogen). Reaction conditions include: denaturation at 95 °C for 2 min, 30 amplification cycles at 95 °C for 10 s, 60 °C for 20 s, and 72 °C for 20 s, and melt curve analysis at 72 °C for 10 min.

### Bioinformatic analysis

RNAseq data was trimmed by Trim Galore using the default Illumina Adapter Sequences. The trimmed reads were mapped to the Ensembl mouse transcriptome GRCm38.73 (mm10) using the default settings of TopHat 2.0.8. The FPKM was then calculated using mapped reads by Cufflinks v2.1.1 under default settings. Analysis of genomic regions surrounding the transcription start site of ELF2 isoforms for putative transcription factor binding sites was performed using MatInspector (Genomatix). Experimentally validated transcription factor binding sites were obtained from UCSC and Ensembl browsers by viewing publicly available ChIPseq datasets. Alignments to determine conservation in genomic DNA and protein sequences were performed using orthologous sequences obtained from Ensembl and aligned using the ClustalW algorithm within MacVector. Prediction of NLS sequences was performed with SeqNLS. Protein disorder analysis was performed using the PONDR server.

### Chromatin immunoprecipitation (ChIP)

For each ChIP, 5 × 10^6^ HEK293T cells transfected with pCCLteteGFP, pCCLteteGFP-2A-HAELF2A, and pCCLteteGFP-2A-HAELF2B were cross-linked with 1% (w/v) formaldehyde for 10 min and were quenched with 1 M glycine to a final concentration of 20 mM. Nuclear lysates were sonicated for 25 cycles, 30 s on, 30 s off using a Bioruptor sonicator (Diagenode). Antibodies for immunoprecipitating protein/DNA complexes include: acetylated H3K9/K14 (#9677, Cell Signaling); CTCF (07-729, Millipore); and HA (ab9110, Abcam). Protein G-conjugated agarose beads (Millipore) were used to immunoprecipitate antibody-bound chromatin complexes, and all subsequent steps were performed according to the manufacturers’ instructions. After de-crosslinking, phenol/chloroform extraction, and ethanol precipitation, PCR was performed on genomic DNA targets using Phusion polymerase with GC buffer (Finnzyme). Primer sequences are in Additional file 1: Table S1.

### Antibody production and purification

Isoform-specific antibodies were raised to recognise the N-termini of ELF2A (aa 2-19) and ELF2B (aa 2-19). Each peptide was synthesised and conjugated to keyhole limpet hemocyanin (KLH) by Mimotopes (Victoria, Australia) and then sent to the Institute of Medical and Veterinary Science (IMVS, Adelaide, Australia) for a series of rabbit immunisations performed according to their standard operating procedures and approved institutional animal ethics protocols. The antiserum collected from the final bleed was used for subsequent affinity purification procedures. Antibodies were purified from crude rabbit serum using thiopropyl sepharose 6B (GE Healthcare) according to the manufacturers’ instructions.

### Western analysis

Cell lysates were prepared using a whole cell lysis buffer (20 mM Tris-Cl pH 7.6, 150 mM NaCl, 1% (v/v) Triton X-100, 0.5% (w/v) sodium deoxycholate, 0.1% (w/v) SDS). Nuclear and cytoplasmic fractionation was performed using the NE-PER Nuclear and Cytoplasmic Extraction Kit (Thermo Scientific), as per the manufacturers’ instructions. Protein samples were denatured at 90 °C for 10 min with 100 mM DTT in NuPAGE® LDS sample buffer. Samples were separated using a 4–12% NuPAGE® Novex® Bis-Tris mini gel (Invitrogen) and transferred onto PVDF membrane (Millipore) using Trans-Blot® SD Semi-Dry Transfer Cell (BioRad). Each blot was then probed with antibodies specific to the protein of interest (Additional file 2: Table S2).

### FACS

To prepare cells for fluorescence-activated cell sorting (FACS), single cell suspensions of cultured cells were filtered to remove cellular debris and aggregates and then were resuspended in 400 μL of PBS containing 2% (v/v) FCS and 5 μg/mL PI. Transduced GFP-positive cells were purified (to > 95% purity) using a BD Influx into sterile 5 mL polystyrene FACS tubes.

### Cell biology assays

For colony-forming assays, FACS-enriched cells were plated in triplicate at 1000 cells/10 cm plate and were incubated for 14 days with media replaced every 5 days. Cells were fixed with 5 mL of ice-cold methanol for 10 min. Plates were air-dried and stained for at least 2 h with Giemsa solution diluted 1:20 in distilled water. Colonies were scored on a digital colony counter (Labserv Technologies). For cellular proliferation assays, FACS-enriched cells were seeded in triplicate wells at 200–1000 cells/well in a 96-well plate in 100 μL of media. Proliferation was assessed every 2 days for a total of 10 days or daily for 4 days. At each time-point, proliferation was measured by MTT assay (Chemicon) according to the manufacturer’s instructions and absorbance was measured by spectrophotometry at 572 nm using a POLARstar Omega microplate reader (BMG Labtech). For cell cycle analysis, BrdU (150 μg/mL diluted in medium) was added to approximately 1 × 10^6^ cells and incubated for 4 h. Cells were rinsed twice in PBS, detached from plates using TrypLE™, fixed, and stained for BrdU incorporation using the APC BrdU Flow Kit (BD Bioscience), following the manufacturer’s instructions. The cells were subsequently incubated in 7-AAD (BD Bioscience) and were analysed on a Canto-II flow cytometer (BD). For cell division analysis, approximately 3 × 10^5^ cells were labelled with 10 μM of CFSE Cell Trace Violet (Invitrogen), according to the manufacturers’ instructions. Following CFSE labelling, cells were resuspended in media and were divided equally into plates containing media with or without doxycycline (1 μg/mL). Cells were allowed to proliferate for 4 days and were subsequently analysed on a flow cytometer. All flow cytometry data was analysed using FlowJo software (Treestar).

### Apoptosis assays

For assessment of Annexin V staining, approximately 1 × 10^6^ cells were labelled with Annexin V reagent conjugated to Pacific Blue or APC fluorophores (BioLegend). Cells were incubated on ice for 1 h, rinsed, resuspended in 200 μL of binding buffer (10 mM HEPES pH 7.4, 140 mM NaCl, 25 mM CaCl_2_) containing PI, and analysed on an LSR Fortessa (BD) flow cytometer. To measure caspase activation, cells were seeded at 1 × 10^4^ cells/well in a 96-well plate and incubated in a 5% CO2-humidified 37 °C incubator for 24 h. Apoptosis was measured using the Caspase Glo 3/7 Assay (Promega) according to the manufacturers’ instructions. The luminescent signal was measured using a POLARstar Omega microplate reader. The luminescent signals measured were normalised to untransduced HeLa control, set as 1.0, for each experiment to account for signal variation between experiments.

### Immunofluorescence staining

HeLa cells were seeded (2 × 10^4^ cells/well) in 8-well chamber culture slides (BD Biosciences) and incubated overnight. The cells were fixed in 4% (w/v) PFA, permeabilised in 0.2% (v/v) Triton X-100, and blocked with 20% (v/v) BlokHen (Aves Laboratories). Cells were then stained with antibodies at optimal dilutions (Additional file 2: Table S2). The cells were stained with DAPI (1 μg/mL) before visualisation using a DM6000 microscope (Leica Microsystems).

### Generation of retrogenic mice and haemopoietic cell analysis

Establishment of GP + E86 NIH3T3-based ecotropic packaging cell lines expressing pMIG retroviral vectors containing eGFP empty vector (control) or HA-tagged ELF2 isoforms was performed as described [28]. Transduction of mouse bone marrow and generation of retrogenic mice was performed as described [28]. For analysis of haemopoietic cell populations, single cell suspensions were stained with relevant antibodies diluted in FACS buffer (PBS + 2% (v/v) FCS) with or without 5 μg/mL propidium iodide (PI). Cells were stored on ice until flow cytometry analysis on an LSR Fortessa (BD). The antibody-fluorochrome conjugates and flow cytometry filter sets used to identify specific haemopoietic populations are described in Additional file 3: Table S3.

### ATRA-induced MPRO differentiation

MPRO cells (2.5 × 10^5^ cells/mL) were treated with 10 μM ATRA. After 72 h treatment, the cells were stained with FITC-conjugated anti-Gr-1 (Ly6G/Ly6C) antibodies (BioLegend), and the cells enriched by FACS for different stages of differentiation based on Gr-1 staining. RNA was isolated from sorted cells for subsequent RT-qPCR analysis. Each FACS-enriched population was cytospun onto glass slides using the Shandon CytoSpin III Centrifuge (GMI), according to the manufacturer’s instructions. May-Grunwald Giemsa staining was performed by the NATA-accredited Haematology Laboratory at Royal Prince Alfred Hospital (Sydney, Australia).

## Results

### Distinct expression of ELF2 isoforms in normal tissues

**Fig. 1:**
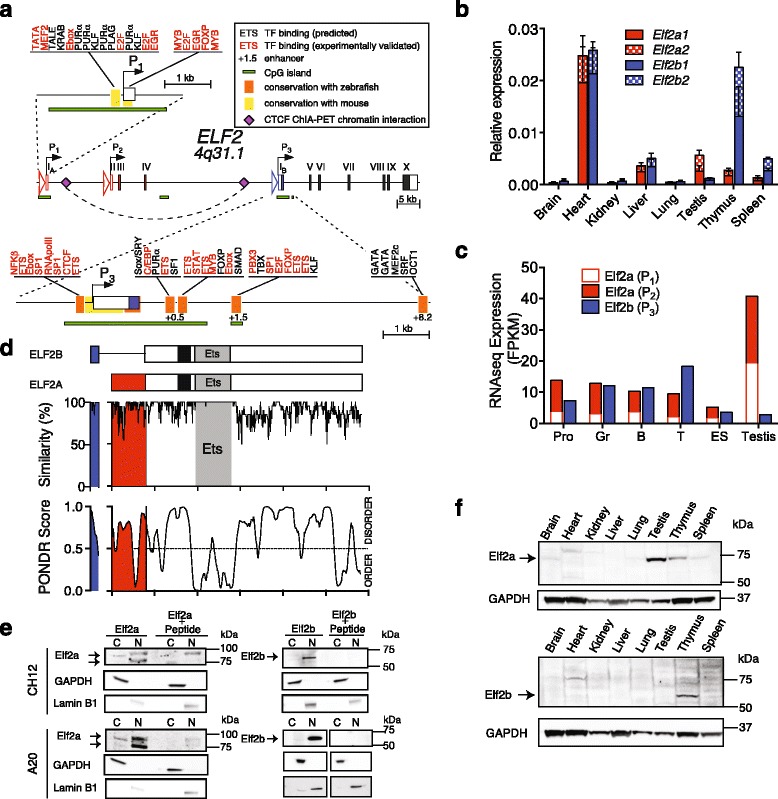
Distinctive expression of ELF2 isoforms in normal tissues. **a** *ELF2* genomic locus indicating isoforms arising from alternative promoters: P_1_ and P_2_ (*ELF2A*) and P_3_ (*ELF2B*). Coding exons unique to *ELF2A* (*red shading*) and *ELF2B* (*blue shading*), common exons (*black shading*) and untranslated regions (*no shading*). Putative enhancer sequences in ELF2B (+0.5, +1.5, +8.2) indicate distance (*in kb*) from the transcription start site in P_3_. Transcription factor binding sites predicted (*black*) and experimentally validated (*red*) are indicated. **b** RT-qPCR analysis of *Elf2a* and *Elf2b* isoforms in mouse tissues normalised to β-actin expression. **c** RNAseq analysis of Elf2 isoforms arising from alternate promoters in mouse tissues: *Pro* promyelocytes, *Gr* granulocytes; *B* B cells, *T* T cells, *ES* embryonic stem cells. Expression level of isoforms arising from each promoter is given in FPKM (fragments per kilobase of transcript per million mapped reads). **d** Schematic of ELF2 protein isoforms: *shading* indicates domains unique to ELF2A (*red*) or ELF2B (*blue*) and the common *Ets* DNA-binding domain (*grey*) and putative bipartite NLS (ELF2A aa 160-190; ELF2B aa 100-130; *black*). A similarity plot of 20 orthologues (human to zebrafish) and PONDR analysis of protein disorder are shown below. **e** Western blot confirming the subcellular localisation of ELF2a in mouse A20 and CH12 B cell lines: *C* cytoplasmic, *N* nuclear lysates. GAPDH and Lamin B1 are positive controls for cytoplasmic and nuclear loading, respectively. Immunising peptide was used to pre-block antibodies where indicated. **f** Western blot of Elf2a and Elf2b expression in mouse tissues

In order to understand how *ELF2* isoform expression is regulated, we first investigated its genomic locus at 4q13.1, which has not been previously characterised. ELF2 isoform expression arises from distinct alternative promoter usage: *ELF2A* expression is driven by promoter P_1_ (starting at exon I_A_) or promoter P_2_ (arising at exon II), but contains identical coding exons; whereas *ELF2B* transcription initiates at promoter P_3_ (starting at exon I_B_) (Fig. 1a). Domains proximal to the regulatory regions of *ELF2A* (P_1_) and *ELF2B* (P_3_) are phylogenetically conserved in mouse. The same domains in *ELF2B* are conserved in zebrafish. We analysed each conserved regulatory region and predicted binding sites for numerous constitutive (Sp1, Ebox, E2F) and haemopoietic-specific transcription factors (ETS, MEF2, GATA, MYB, FOXP, NFKß, and C/EBP). Many have been experimentally verified in chromatin immunoprecipitation (ChIP) studies and were identified in putative conserved enhancer regions (+0.5, +1.5, and +8.2) proximal to P_3_ (*ELF2B*) (Fig. 1a). HA-tagged cDNAs encoding human ELF2A and ELF2B were cloned into eGFP-containing vectors and transfected into HEK293T cells to confirm whether they could bind *Ets* sites by ChIP (Additional file 4: Figure S1A, B). ChIP PCR confirmed ELF2A binding to *VCP*, *PYGO2*, *LMO2,* and *LYN* promoters, whereas ELF2B was only detected binding *PYGO2* and *VCP* promoters (Additional file 4: Figure S1C). Within the regulatory regions of ELF2, we observed binding of ELF2A and ELF2B to regions downstream of *ELF2A* P_1_ and *ELF2B* P_3_ promoters (*ELF2A* +0.5 and *ELF2B* +1.5, respectively), suggesting that ELF2 isoforms are able to auto-regulate their own expression (Additional file 4: Figure S1C).

We next designed RT-qPCR primers to quantitate all Elf2 isoforms: including major isoforms *Elf2a1* (NERF-2a) and *Elf2b1* (NERF-1a) and minor isoforms, *Elf2a2* (NERF-2b) and *Elf2b2* (NERF-1b), which arise from alternative splice acceptor usage at exon VI leading to inclusion of an extra 36 bp of intronic sequence (Additional file 5: Figure S2A). Each isoform amplicon was independently verified by Sanger sequencing (Additional file 5: Figure S2B). We first examined the expression of Elf2 isoforms by RT-qPCR in various C57BL/6 mouse tissues (Fig. 1b). Each Elf2 isoform is expressed in equivalent abundance in the brain, heart, kidney, liver, and lung consistent with previous reports [23]. However, we note for the first time that *Elf2b* is preferentially expressed in the thymus and spleen whilst *Elf2a* is preferentially expressed in the testis (Fig. 1b). We then analysed the expression of each Elf2 isoform in various mouse haemopoietic cells or tissues from in-house [29] or publicly available RNAseq data (Fig. 1c). These data suggest that *Elf2a* is preferentially expressed in the testis compared to *Elf2b*, whilst *Elf2b* is generally expressed at higher abundance in lymphoid tissues (thymus and spleen), consistent with our RT-qPCR data. Elf2 isoform expression analysis in a range of mouse haemopoietic cell lines also indicated a preference for *Elf2b* expression over *Elf2a* in lymphoid cell lines (Additional file 5: Figure S2C). In nearly all tissues and cell lines, the major isoforms of Elf2 were expressed more abundantly than the alternatively spliced minor isoforms (Fig. 1b, Additional file 5: Figure S2C), thus only the major Elf2 isoforms are examined in the remainder of this study.

Comparison of the amino acid sequence similarity in ELF2 orthologues illustrated a high level of conservation in the Ets domain (Fig. 1d). The N-termini of ELF2A and ELF2B were also highly conserved (91.5 and 98.2% similarity, respectively), indicating they are both functionally important. Both N- and C-termini are also intrinsically disordered (Fig. 1d) indicating these regions may be important for recruiting binding partners. We next raised ELF2 isoform-specific antibodies against both N-termini to measure protein expression (Additional file 5: Figure S2D). Antibodies were affinity purified with their respective immunising peptides, and subsequently validated to be isoform-specific and cross-react with mouse and human ELF2 proteins (Additional file 5: Figure S2E). We also showed Elf2 isoforms were predominantly nuclear localised (Fig. 1e) in mouse CH12 and A20 B lymphoma cell lines, however, some Elf2a was also detectable in the cytoplasmic fraction. In CH12 and A20 cells, two Elf2a species of differing molecular weights were detected in the nuclear fraction consistent with post-translational modification by phosphorylation [30]. Elf2a protein is most abundantly expressed in testis, followed by thymus and spleen, consistent with our expression data (Fig. 1b, c, f). Elf2b protein was abundant in the thymus suggestive of a role in T cell development (Fig. 1f) and confirm expression data. Here, we have demonstrated that the Elf2 isoforms have distinct expression in different tissue and cell types. This differential expression may impact on the regulation of ELF2 targets in a tissue-specific manner.

### ELF2B overexpression decreases cellular proliferation and clonogenicity

**Fig. 2:**
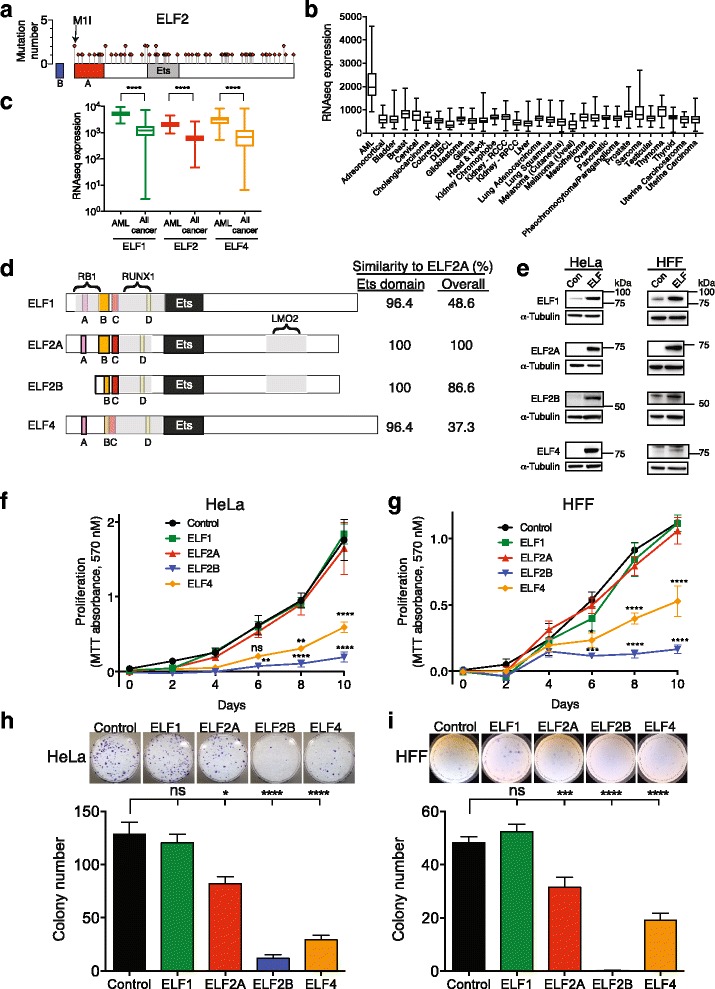
ELF2B overexpression decreases cellular proliferation and clonogenicity in vitro. **a** Number and distribution of somatic mutations in ELF2A and ELF2B compiled from TCGA and COSMIC databases (see Additional file 6: Table S4). **b** RNAseq expression analysis of ELF2 from 30 TCGA studies; data is represented with *box* and *whisker plots*, showing quartiles and minimum and maximum values. Expression is in RNASeq V2. **c** RNAseq expression of ELF subfamily members in acute myeloid leukaemia (AML) compared to all other cancers (29 in total) from TCGA data. Expression is in RNASeq V2 (log). **d** Schematic of ELF family members showing the conserved *Ets* DNA-binding domain, conserved acidic domains A–D and known protein interaction domains for RB1, RUNX1, and LMO2. Amino acid similarity scores between ELF2A and all ELF proteins are indicated. **e** Overexpression of ELF proteins in HeLa and HFF cells: with control (*GFP empty vector*, *Con*) and HA-tagged ELF protein-containing lentivectors. MTT proliferation assay in HeLa (**f**), and HFF cells (**g**). Clonogenicity assay in HeLa (**h**) and HFF cells (**i**). Representative images of Giemsa-stained colonies are shown. Data represents the mean ± SEM of three experiments each performed in triplicate with statistical analysis performed using Mann-Whitney *U* test (*ns*, not significant; *, *p* < 0.05; **, *p* < 0.01; ***, *p* < 0.001; ****, *p* < 0.0001). Statistical significance is indicated relative to GFP control

As the potential role of ELF2 in cancer has not been explored, we analysed ~150 cancer genome sequencing cohorts deposited with The Cancer Genome Atlas (TCGA) and the Catalogue of Somatic Mutations in Cancer (COSMIC) for ELF2 mutations and expression. In over 5000 patient samples, 77 somatic mutations were distributed evenly throughout *ELF2* (Fig. 2a, Additional file 6: Table S4). Interestingly, an M1I non-start missense mutation in two cancer samples would abrogate ELF2A expression, resulting in ELF2B expression only (Fig. 2a). Analysis of RNAseq data from 30 cancer studies revealed that *ELF2* was more highly expressed in acute myeloid leukaemia (AML) than any other cancer (Fig. 2b). Comparison of other ELF family members showed ELF1 and ELF4 were also more highly expressed in AML than all other cancers, suggesting that they may play a role in AML (Fig. 2c). The haemopoietic-specific ELF1, ELF2A, and ELF4 have very similar *Ets* DNA-binding domains, but exhibit less amino acid similarity within their termini (Fig. 2d). However, these ELF proteins are distinguished by the presence of homologous acidic domains ‘A’, ‘B’, ‘C’, and ‘D’ [23, 24] in their N-termini (Fig. 2d). Complete acidic domains A and B, which have transactivation activity, are absent in ELF2B. All ELF family members, however, interact with RUNX1 through their N-termini [24, 31]. Both ELF2A and ELF2B uniquely interact with the haemopoietic transcriptional co-regulator and proto-oncogene LMO2 [22] whilst ELF1 specifically interacts with the tumour suppressor RB1 [32] (Fig. 2d).

To characterise the functional role of ELF2 isoforms, we overexpressed ELF1, ELF2A, ELF2B, and ELF4 in human primary and immortalised cells using a doxycycline (Dox)-inducible lentivector (Additional file 4: Figure S1A). We chose the most suitable cell lines by examining endogenous expression of each ELF protein (Additional file 7: Figure S3A). HeLa and primary human foreskin fibroblasts (hFF) were found to express the lowest overall level of each ELF protein and were thus chosen for this study (Additional file 7: Figure S3A). Overexpression of ELF proteins after lentiviral transduction was confirmed by western blot analysis (Fig. 2e), and was shown to be nuclear localised by immunofluorescence staining (Additional file 7: Figure S3B).

The proliferative ability of ELF protein-transduced HeLa and hFF cells was then assessed by MTT assay. ELF2B and ELF4 overexpression significantly reduced cellular proliferation in both HeLa and hFF cells compared to control (eGFP only-expressing cells) (Fig. 2f, g, *p* < 0.0001) with ELF2B showing the most dramatic inhibition. Overexpression of ELF1 or ELF2A did not affect proliferation in either HeLa or hFF cells compared to control. To assess the effects of ELF protein overexpression on the clonogenic capacity of hFF and HeLa cells, cells were plated at low density in a colony-forming assay following FACS enrichment. Cells overexpressing ELF2B displayed the most profound reduction in clonogenic ability compared to control cells (HeLa: *p* < 0.0001 and hFF: *p* < 0.0001) followed by ELF4 (HeLa: *p* < 0.0001 and hFF: *p* < 0.0001) (Fig. 2h, i). ELF2A overexpression also decreased the clonogenic ability of HeLa cells (*p* = 0.0242) and hFF cells (*p* = 0.007) whereas ELF1 overexpression had no effect.

### ELF2B protein overexpression minimally disrupts cell cycle kinetics

**Fig. 3:**
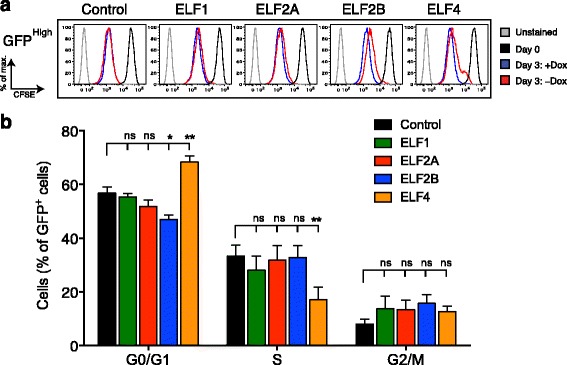
ELF2B overexpression minimally disrupts cell cycle progression. **a** CFSE-labelled high GFP^+^ (GFP^High^) HeLa cells expressing ELF proteins were incubated ± Dox for 3 d. **b** Cell cycle analysis of ELF protein-expressing HeLa cells using BrdU incorporation. Data in **b** represents the mean ± SEM of three experiments each performed in triplicate with statistical analysis performed using Mann-Whitney *U* test (*ns*, not significant; *, *p* < 0.05; **, *p* < 0.01)

As ELF2B significantly curtailed cellular proliferation in primary and immortalised cells, we next determined whether its overexpression affected cell cycle progression. HeLa cells overexpressing ELF proteins were enriched by FACS and stained with CFSE (Additional file 8: Figure S4A). Cells with high, medium, and low GFP expression or the bulk GFP^+^ population were grown in the presence or absence of Dox to regulate ELF protein expression (Fig. 3a and Additional file 8: Figure S4B, C). The bulk GFP^+^ ELF2B and ELF4 cells grown in the absence of Dox underwent less cell division as indicated by higher CFSE staining, whereas ELF1 and ELF2A were identical to controls (Additional file 8: Figure 4B). Only with high ELF2B- and ELF4 expression did we observe a pronounced delay in cell division (Fig. 3a), whereas there were minimal effects in low and medium-expressing populations (Additional file 8: Figure S4C). We next performed cell cycle analysis using BrdU incorporation in ELF-expressing HeLa cells to establish which cell cycle stages were affected (Additional file 8: Figure S4D). ELF2B expressing cells exhibited only a decrease in the G0/G1 population (*p* = 0.011). ELF4 expressing cells, in contrast, accumulated in G0/G1 phase (*p* = 0.0041) with a concomitant decrease in S phase (*p* = 0.0041) (Fig. 3b). This distinctly different disruption of cell cycle kinetics by ELF2B compared to ELF4 is likely due to the regulation of distinct *Ets* target genes or the inhibition of binding of target sites by other *Ets* factors.

**Fig. 4:**
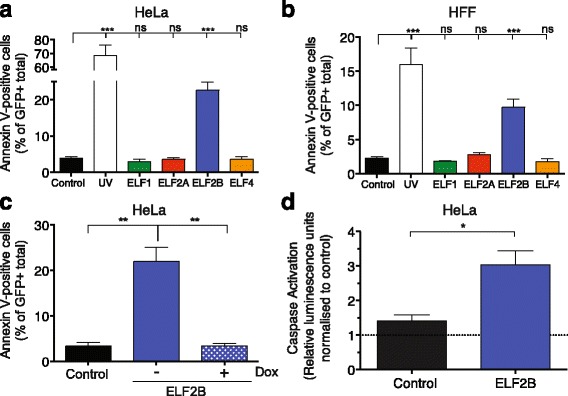
ELF2B overexpression induces apoptosis in vitro. Annexin V-labelling of HeLa (**a**) and HFF cells (**b**) overexpressing ELF proteins; cells recovered for 18 h after UV exposure were included as a positive control. **c** Annexin V assay of ELF2B expressing cells incubated ± Dox for 3 d. **d** Caspase 3/7 activation assay performed in HeLa cells normalised to non-transduced HeLa control cells; statistical analysis is performed compared to GFP control. Data represents the mean ± SEM of three experiments with statistical analysis performed using Student’s *t* test (*ns*, not significant; *, *p* < 0.05; **, *p* < 0.01; ***, *p* < 0.001)

### ELF2B overexpression induces apoptosis in vitro

We observed that distinct morphological changes occurred in ELF2B- and ELF4-overexpressing cells (Additional file 8: Figure 4E). Most cells no longer expressed GFP, were shrivelled and crenated in appearance, and lacked membrane integrity—indicative of cell death. Given the reduced cellular proliferation and the changes in morphology observed in cells, we propose that overexpression of ELF2B or ELF4 may induce apoptosis. To confirm this, annexin V- and PI-staining was performed on HeLa and hFF cells transduced with control or ELF protein-containing vectors or subjected to UV insult as a positive control. Annexin V-positive cells were only detected in UV-treated and ELF2B-overexpressing HeLa and HFF cells (Fig. 4a, b; *p* < 0.001). This induction of apoptosis in HeLa cells was abrogated when ELF2B expression was suppressed by the addition of Dox (Fig. 4c; *p* = 0.004). A caspase activation assay was performed to confirm whether ELF2B expression induced apoptotic cell death. Similar to previous observations, ELF2B overexpression resulted in ~twofold increase in activated caspase levels compared with control (Fig. 4d, *p* = 0.021).

### The N-terminus of ELF2B has repressor activity

**Fig. 5:**
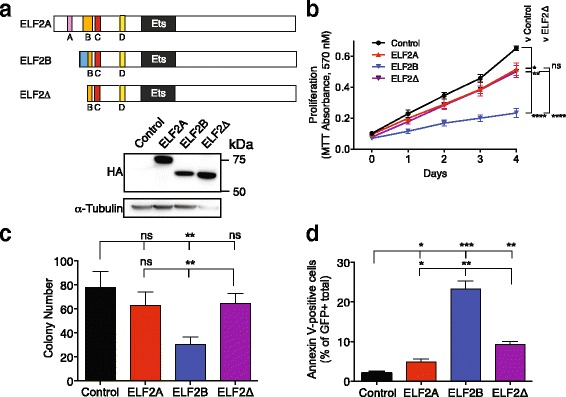
ELF2B’s repressor function is conferred by its N-terminus. **a** Schematic of ELF2Δ and confirmation of expression in HeLa cells by western blot. HeLa cells transduced with ELF2 isoforms and ELF2Δ were analysed by MTT (**b**), clonogenicity (**c**), and Annexin V apoptosis assays (**d**). Data represents the mean ± SEM of three experiments with statistical analysis performed using Student’s *t* test (*ns*, not significant; *, *p* < 0.05; **, *p* < 0.01; ***, *p* < 0.001; ****, *p* < 0.0001)

As ELF2B is functionally distinct from ELF2A in overexpression studies, we next examined whether the presence of the ELF2B N-terminus accounted for the phenotypic differences observed. To address this, a 525 aa truncated form of ELF2 (ELF2∆) which lacks any isoform-specific N-terminal sequence and has an expected 56 kDa molecular weight was generated and then verified by western blot (Fig. 5a). Immunofluorescence staining using anti-HA antibodies demonstrated that ELF2∆ was similarly nuclear-localised to ELF2A and ELF2B (Additional file 7: Figure S3B). Overexpression of ELF2∆ in HeLa cells only slightly decreased cellular proliferation compared to control (*p* = 0.003) and to levels equivalent to ELF2A, but significantly reversed the anti-proliferative effect of ELF2B (*p* < 0.0001, Fig. 5b). Similarly, deletion of ELF2B’s N-terminus abrogated the suppression of colony-forming capacity by ELF2B (*p* = 0.008), back to levels equivalent to ELF2A and control (Fig. 5c). These data suggest that sequences within the 19 aa N-terminus of ELF2B are required for the dominant negative effects of ELF2B. As overexpression of ELF2B-induced apoptosis, we determined the effect of deleting the N-terminus on ELF2B function. Annexin V and PI staining was performed on HeLa cells transduced with control, ELF2A, ELF2B, or ELF2∆. As expected, ELF2B overexpression resulted in a ~11-fold increase in apoptotic activity compared with control (*p* = 0.0005) (Fig. 5d). However, ELF2∆ overexpression resulted in only a ~fourfold increase in apoptotic activity compared to control (*p* = 0.0012), which was an intermediate effect compared to ELF2A (*p* = 0.0168) and ELF2B (*p* = 0.003) (Fig. 5d). Thus, deletion of N-terminal isoform-specific domains of ELF2 can also alter the cellular response to apoptosis induction.

### ELF2A and ELF2B are regulators of early lymphocytic development

ELF2 is widely expressed in haemopoietic tissues and cell lines and transcriptionally regulates genes involved in early B and T cell development including the signal-transducing Src-family of receptor tyrosine kinases such as BLK, LYN, and LCK and immunoglobulin enhancers (Additional file 9: Table S5). We generated retrogenic mice expressing ELF2 isoforms to determine their individual contribution to haemopoietic development and differentiation. To perform this, we used murine leukaemia virus (MLV) retroviral vectors containing HA-tagged Elf2 isoforms and GFP (Additional file 10: Figure S5A) with a murine bone marrow reconstitution model. Haemopoietic progenitor cells were isolated, transduced, and then transplanted into sublethally irradiated recipient *Rag1*-deficient mice. Sustained Elf2 overexpression was confirmed by RT-PCR analysis of splenocytes after 3 months (Additional file 10: Figure S5B). Analysis of peripheral blood mononuclear cells at 4 weeks post-transplant indicated that ~43% cells were transduced with control GFP vector and ~3–8% cells were marked with Elf2 isoform-containing vectors (Additional file 10: Figure S5C). We observed a significant change in retrogenic peripheral T cells with a significant decrease in the CD4:CD8 ratio in Elf2b isoform-expressing cells (1.30 in control vs 0.79 and 0.82 in Elf2b isoforms; Additional file 10: Figure S5D). We observed an increase in the number of peripheral B220^+^ B cells in Elf2a-expressing cells (Additional file 10: Figure S5E). Changes were not observed in mature granulocyte numbers (Additional file 10: Figure S5F). At 3 months post-transplant, the haemopoietic compartment (thymus, spleen, bone marrow, and peritoneum) showed stable reconstitution (Additional file 10: Figure S5G). These data indicate that although the level of gene marking of reconstituted cells was low, possibly due to repression of the MLV promoter by Elf2 at a known *Ets* DNA binding site [33], ectopic Elf2 isoform expression was still able to perturb lymphocytic development and differentiation in reconstituted mice. These data are suggestive of a role for Elf2a and Elf2b in B and T cell development. As similar observations were made for major and minor isoforms of both Elf2a and Elf2b (Additional file 1: Figure S5C–G), only data for the major isoforms Elf2a1 and Elf2b1 are presented herein.

**Fig. 6:**
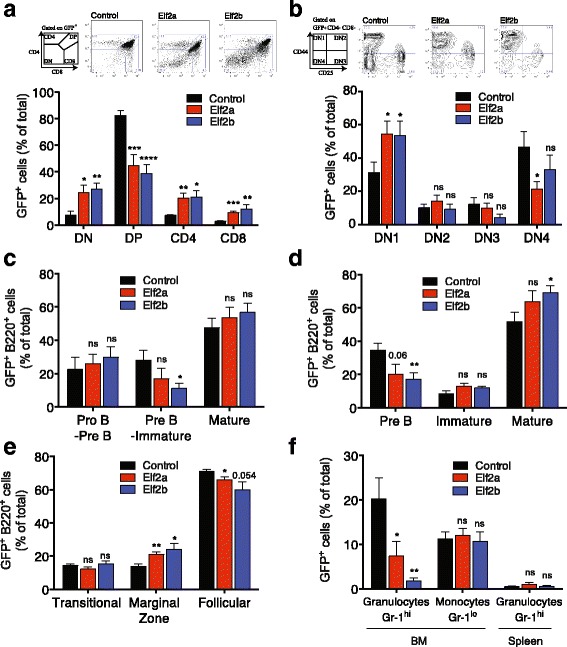
ELF2 isoform expression affects early lymphocytic development. Analysis of ELF2^+^ (*GFP* ^*+*^) retrogenic ‘double negative’ (*DN*), ‘double positive’ (*DP*) or single positive CD4^+^ and CD8^+^ T cells (**a**), and DN cells at each developmental stage in the thymus (**b**); representative flow cytometry plots indicating perturbation of the T cell compartment are shown (**a**–**b**). Analysis of ELF2^+^ (*GFP* ^*+*^ *)* retrogenic B cells in the bone marrow stained with CD43 and B220^+^ (**c**) and with IgM and B220^+^ (**d**). Analysis of B cell maturation in the spleen (**e**), and myeloid subpopulations in bone marrow (*BM*) and spleen (**f**), of ELF2^+^ retrogenic mice. Data represents the mean ± SEM of three experiments each performed with 4–5 mice per experimental arm. Statistical analysis performed using Student’s *t* test (*ns*, not significant; *, *p* < 0.05; **, *p* < 0.01; ***, *p* < 0.001)

Examination of T cell development in the thymus of Elf2-overexpressing mice revealed a significant two to threefold increase in the number of GFP^+^ double-negative (DN) thymocytes compared to control (Fig. 6a). There was a concomitant 30–60% reduction in DP T cells (*p* < 0.001), and approximately threefold increase in mature CD4^+^ and CD8^+^ T cells compared with control (*p* < 0.05) (Fig. 6a). Analysis of the early committed DN T lymphocyte population showed a ~twofold increase in DN1 T cells compared to control (*p* < 0.05) (Fig. 6b), and a ~twofold reduction in DN4 T cells in Elf2a-overexpressing mice (*p* < 0.05). A similar trend was observed in Elf2b-overexpressing mice, which did not reach statistical significance. DN2 and DN3 stages were unaffected. To further support these findings, the expression of TCRβ was examined in the thymus of reconstituted mice. Early committed T cells lack expression of TCRβ, cells in DN2 to DN4 stages express low levels of TCRβ, whilst TCRβ expression is highest in mature CD4^+^ and CD8^+^ T cells. In mice overexpressing Elf2 isoforms, there were a higher percentage of TCRβ^hi^ T cells (*p* < 0.05), whilst a reduction was observed in TCRβ^lo^ T cells (*p* < 0.01) (Additional file 11: Figure S6A). This supports our earlier findings that Elf2 overexpression caused a perturbation in T cell development, with a decrease in DP T cells and an increase in mature CD4^+^ and CD8^+^ T cells in the thymus. Examination of mature TCRβ^hi^ lymphocytes in the spleen did not reveal changes in the numbers or proportions of CD4^+^ or CD8^+^ T cells (Additional file 11: Figure S6B, C). Further investigation of CD4^+^ and CD8^+^ subsets, including naïve and memory CD4^+^ T cells (Additional file 11: Figure S6D), CD8^+^ naïve, effector and central memory cells (Additional file 11: Figure S6E), and CD4^+^ regulatory T cells (Additional file 11: Figure S6F) similarly showed no changes resulting from Elf2 overexpression.

Examination of the developing B cell subsets in the bone marrow using CD43 and B220 surface markers indicated that Elf2b-expression decreased the percentage of pre-B to immature B cells compared with control (Fig. 6c; *p* < 0.05); a similar decrease was also observed in Elf2a-expressing cells, which did not reach statistical significance (Fig. 6c). Further analysis using IgM and B220 surface markers revealed that the pre-B cell population, rather than the immature B cell population, was reduced by approximately 50% in Elf2b-expressing cells (*p* < 0.01, Fig. 6d). A similar decrease was observed in Elf2a-expressing cells, albeit not reaching significance (*p* = 0.06) (Fig. 6d). An increase of ~10–25% was also observed in mature recirculating Elf2b-transduced B cells, however, this may be due to the reduced distribution of pre-B cells (Fig. 6d). Examination of B cell maturation in the spleen revealed an increase of approximately 50% in marginal zone B cells transduced with Elf2a and Elf2b, and a concomitant small decrease in follicular B cells, whilst no changes were observed in transitional (T1 and T2) B cells (Fig. 6e).

We next examined the myeloid compartment in reconstituted mice by analysing bone marrow cells and splenocytes stained with Gr-1. A 3–12-fold reduction in the Gr-1^hi^ granulocytic population was observed in the bone marrow of Elf2a- and Elf2b-overexpressing mice (*p* < 0.05) (Fig. 6f). The Gr-1^lo^CD11b^+^ monocytic population in the bone marrow remained unchanged after Elf2 overexpression (Fig. 6f). To further examine the potential role of Elf2 on granulocyte maturation, we used the MPRO mouse promyelocytic cell line that can be induced to differentiate into mature granulocytes with all-*trans* retinoic acid (ATRA) [34]. MPRO cells were treated with ATRA for 72 h to induce myeloid differentiation and then FACS enriched based on Gr-1 expression (Additional file 12: Figure S7A). The morphology of each differentiated population was confirmed (Additional file 12: Figure S7B), and key genes regulated during granulocytic differentiation were assessed by RT-qPCR (Additional file 12: Figure S7C). Our qPCR analysis confirmed gene signatures of granulocytic differentiation (Additional file 12: Figure S7C). These included *Ctsg* (primary granule; downregulated), *Ltf* (secondary granule; upregulated), and *mmp9* (tertiary granule; upregulated). We analysed Elf2 isoform expression in each population and showed that *Elf2b* expression was significantly decreased by twofold in mature granulocytes (Gr-1^hi^) compared to promyelocytes (Gr-1^lo^) (*p* < 0.01, Additional file 12: Figure S7D); with the same trend observed for *Elf2a1* expression (*p* = 0.053). This indicates that *Elf2* downregulation may be important in permitting the final stages of granulocyte maturation and compliments our observation that Elf2 overexpression inhibits granulocytic differentiation.

## Discussion

Historically, distinct ELF2 isoforms were described (NERF-1a, NERF-1b, NERF2) with some functional attributes [22–24, 35, 36]. However, more recent studies on ELF2 function have been challenging to interpret as the exact ELF2 isoform used in overexpression studies, targeted in knockdown studies or detection methods were often not specified [37–39]. ELF2 isoforms arise from distinct conserved loci, suggesting they may have evolved to play specific functional roles. The unique antibody reagents we developed enabled us to distinguish whether either ELF2 isoform is mutually or exclusively expressed. ELF2A is the major isoform expressed in the testis, whilst ELF2B is preferentially expressed in the thymus. The exact role ELF2 plays in different tissues may be impacted by N-terminal functional differences between isoforms and their proportionate expression. In a similar manner, three OCT1 isoforms that differ at their N-termini, can elicit variable transactivation of the same target genes and can control a different but overlapping set of target genes [40].

A distinguishing feature of ELF subfamily members as opposed to other Ets proteins is the centrally positioned *Ets* DNA-binding domain, and the intrinsically disordered N and C termini that flank it. Intrinsically disordered regions are more prone to forming protein interaction scaffolds or undergo post-translational modifications [41]. Typically, intrinsically disordered proteins can also form central interaction hubs in signalling pathways [42]. Deletion mutant studies have identified an acidic transactivation domain in ELF1 [43], ELF4 [44], and ELF2A [24]. The lack of an intact transactivation domain in ELF2B supports the functional differences in ELF2B we observed. ELF2B’s unique and conserved N-terminus may interfere with normal ELF2 protein-protein interactions or may recruit unique binding partners that augment its inhibitory function. Deletion of this 19 aa N-terminal domain abrogates ELF2B’s inhibitory function, reversing the anti-proliferative and apoptosis-inducing effects of ELF2B.

Our comprehensive functional analysis of ELF subfamily members was necessary due to the similarities in their *Ets* domain and structure. ELF1, ELF2, and ELF4 all have haemopoietic-specific expression, considerable redundancy in DNA binding [8], and some common binding partners. As a result, ELF family members may compete for binding or exhibit a co-ordinated transactivation program in a stage- or temporally specific manner. ELF2 is able to competitively inhibit ELF1 transactivation of *Tie1* and *Tie2* target sites in chicken blood vessels [45]. Other *Ets* factors can compete with ELF1 for high affinity *Ets* sites, but low affinity sites were still available for ELF1 binding in co-operation with other co-factors [46]. With redundancy in DNA binding between *Ets* factors, binding partners may significantly influence site preference for individual *Ets* proteins in a context-specific manner.

Both ELF2B and ELF4 dramatically reduced cellular proliferation and clonogenicity in primary and transformed cells, whilst ELF1 and ELF2A had negligible effects. Antiproliferative functions have previously been suggested for ELF transcription factors, particularly for the candidate tumour suppressor protein ELF4 [37, 47]. ELF2 (and ELF1) overexpression also resulted in reduced cellular proliferation in transformed cells (T3M-1 CI-10, HT1080, and MCF10A) [37]. However, in contrast to these studies, ELF2 overexpression in hepatoma cells actually enhanced tumour cell proliferation, whilst conversely ELF2 knockdown repressed cell growth [38]. Our data clearly reaffirms the antiproliferative effects of ELF2B and ELF4 and attributes a proapoptotic function to ELF2B.

The disparate effects on cell cycle kinetics observed between ELF2B and ELF4 may arise from differential DNA occupancy. ELF2 knockdown in ES cells resulted in up- or downregulation of fewer than 100 genes [48], whilst ELF4 overexpression in T3M-1 CI-10 cells revealed 95 strongly regulated genes implicated in G1 cell cycle phase regulation and apoptosis [37]. Previous studies have linked a role for ELF4 in cell cycle kinetics with transactivation activity largely limited to the G1 phase [49]. ELF4 regulates the quiescent state of haemopoietic stem cells by facilitating their transition from G0 to G1 [21]. ELF4 overexpression induced an accumulation in the G1 cell cycle stage, consistent with a previous report [37]. Knockdown of ELF2 in SK-Hep1 cells led to an accumulation of cells in G1 using shRNAs that target both ELF2 isoforms [38]. Consistent with these data, ELF2B overexpression resulted in a decrease in G1 phase, but this may have resulted from loss of cells due to apoptosis. As ELF2B can bind *Ets* sites and therefore compete with ELF2A, it may be acting as a dominant negative protein by preventing canonical activation of growth-promoting ELF2A targets. As a consequence of inhibiting normal ELF2A activation, growth suppression and apoptosis may result. Furthermore, as a dominant negative protein, ELF2B may also recruit co-repressors or disrupt protein complexes that normally interact with ELF2A. ELF2B acts as a putative tumour suppressor protein in its ability to decrease proliferation, clonogenic capacity, and induce apoptosis in vitro, however, its ability to inhibit tumour formation in vivo needs to be definitively tested.

Elf2 overexpression drastically affected early T cell development in the thymus, but not peripheral T cells. We observed a significant accumulation of TCRβ^+^ immature single positive (ISP) T cells and concomitant reduction in DP T cells in reconstituted mice after overexpression of ELF2 isoforms. After recombination at the TCRα locus, thymocytes assemble the mature TCR and co-express the co-receptor proteins CD4 and CD8 to form the pool of DP αβ-TCR expressing immature thymocytes, which constitute ~90% of the lymphoid compartment [50]. The decrease in immature DP cells is reflected in an overall decrease in the proportions of surface TCRβ^lo^-expressing cells. Further analysis of ISP DN cells attributes this to an accumulation of DN1 thymocytes. Given the significant decrease in DN4 and DP cells, we postulate that the increase of DN1 immature thymocytes results from an increased T cell lymphoid progenitor recruitment from the bone marrow to compensate for the reduced DP thymocyte output.

We postulate that ELF2 overexpression perturbs T cell development by interfering with pre-TCR assembly and activation. This could arise via interference with the expression of co-receptors, specialised adaptor molecules, and transcriptional regulators. Regulation of apoptosis is key in promoting survival of DN4 and DP thymocytes during pre-TCR signalling and subsequent positive and negative selection following engagement of self-antigen with major histocompatibility complex (MHC)-peptide ligands. Therefore, we also propose that Elf2 overexpression in T cells may impact on negative selection of DP thymocytes, leading to subsequent decreased DN4 and DP populations. For example, mice deficient in *Ets1*, a regulator of pre-TCR signalling, have an impaired development of DN3 to DP cells, which is coupled with increased apoptosis but normal cell proliferation [51]. Similar to Elf2 overexpression, this also resulted in reduced DN4 and DP populations.

During B cell development, common lymphoid progenitors in the bone marrow differentiate into pro-B cells and then transition to pre-B cells. The progression from pro-B cells to pre-B cells involves pre-B cell receptor (BCR) rearrangement via V(D)J recombination. Failure to assemble the BCR complex results in cell death at the first checkpoint in B cell development. In B cells, V(D)J recombination involves an orchestrated cleavage, rearrangement, and joining of DNA segments, which is tightly linked to the cell cycle, particularly in G0 and G1 phases [52]. As Elf2b overexpression in particular reduces the number of cells in G1 phase, this may explain the reduction in precursor B cells progressing to pre-B cells, suggesting pre-BCR development is directly impacted. Alternatively, Elf2 may compete with other *Ets* factors for binding at *Ets* sites, which consequently affect the survival, cell cycle, or DNA rearrangement of pre-B cells. Elf2 also binds IgH enhancers π and μB, which are involved in V(D)J recombination [23, 53] as does ELF1 and PU.1 [54, 55]. ELF2 also regulates the expression of specialised adaptor molecules required for signal transduction after B cell activation, such as BLK and LYN [24], as well as components of the BCR complex such as Igα and Igβ [23].

In this study, we have demonstrated that ELF2B reduces cell proliferation, colony-forming ability, cell cycle progression, and survival. This has an impact in vivo with ELF2 isoforms disrupting the tightly regulated development of B and T cells. The significant reduction in DN4 and DP T cell populations in the thymus and in the pre-B cell population in the bone marrow of mice overexpressing Elf2 is consistent with disruption of key developmental checkpoints. Developing lymphocytes produce specific B and T cell receptors through V(D)J gene rearrangement and recombination events, a process crucial in generating receptor diversity. A functional receptor will confer cell survival and proliferation signals that enable these lymphocytes to progress in their development, whilst failure to form a functional receptor will trigger apoptosis in the developing lymphocyte. ELF2 may therefore play an important role in regulating key effectors involved in V(D)J gene rearrangement for TCR and BCR assembly in early lymphocytic development.

## Conclusions

Our study highlights the importance of specifying which ELF2 isoform is being examined in any future studies involving ELF2. Given the known opposing effects of ELF2A and ELF2B on target gene expression and our evidence of the putative dominant negative functions of ELF2B, we postulate that the interplay between ELF2 isoforms and other related Ets factors may be critical in regulating early lymphocytic development. Although our in vitro studies clearly distinguish between ELF2A and ELF2B function, the phenotypic changes observed in our ELF2^+^ retrogenic mouse models were similar between isoforms. Direct competition of ELF2 isoforms with other *Ets* factors, due to redundancy in occupancy at lymphoid-specific *Ets* sites, may facilitate the perturbation of early lymphocytic development we observed. Further studies should clarify the similarities and differences in ELF2 isoform DNA occupancy and function in vitro and examine the organismal-wide role of ELF2 isoforms in co-ordinating transcription in vivo.

## Additional files

Additional file 1: Table S1. PCR primers used in this study (DOC 69 kb)

Additional file 2: Table S2. List of primary and secondary antibodies used in immunofluorescence (IF) and western blot (WB) analysis. All antibodies were diluted to their working concentrations in the appropriate blocking solution (DOC 43 kb)

Additional file 3: Table S3. Antibody-fluorophore conjugates and filter combinations used to distinguish haemopoietic-specific cell surface markers (DOC 40 kb)

Additional file 4: Figure S1. Confirmation of DNA binding of ELF2 isoforms by ChIP. A) The doxycycline-regulatable ‘dox-off’ lentiviral vector used to co-express eGFP and ELF2 isoforms. B) Flow cytometric analysis of HEK293T cells transfected with eGFP only (control)-, HA-ELF2A- and HA-ELF2B-containing vectors. C) ChIP PCR of known ELF2 targets (*VCP*, *PYGO2*, *LMO2*, and *LYN* promoters), novel ELF2-binding sites in *ELF2* promoter regions (P_1_, P_2_, and P_3_) as well as a negative control region spanning *H19* exons 4 and 5. (PDF 1124 kb)

Additional file 5: Figure S2. Validation of reagents used to detect ELF2 isoform expression. Design A) and validation B) of RT-qPCR primers used to detect Elf2a and Elf2b major and minor isoforms with expected amplicon sizes (bp). C) RT-qPCR detection of Elf2 isoform expression in murine haemopoietic cell lines. D) Specific N-terminal sequences used as immunising peptides to produce isoform-specific antibodies. The amino acid identity between mouse and human sequences is shown. E) Validation of specificity and species cross-reactivity of ELF2A and ELF2B antibodies in control-transduced (GFP vector only; Con) HEK293T cells and cells transduced with mouse Elf2A (mA), mouse Elf2b (mB), human ELF2A (hA), or human ELF2B (hB)-containing lentiviral vectors (PDF 1535 kb)

Additional file 6: Table S4. Somatic mutations in ELF2 in cancer. Mutations are compiled from the TCGA CBIO portal and COSMIC databases. Mutations for ELF2A are shown; no mutations in ELF2B’s 19 aa N-terminus have been recorded (DOC 99 kb)

Additional file 7: Figure S3. Confirmation of ELF protein expression in vitro. A) Determination of endogenous ELF family protein levels in immortalised and primary cells; Con = HeLa cells overexpressing the respective HA-tagged ELF protein. Numbers indicate molecular weight markers (in kDa). B) Confirmation of subcellular localisation of ELF family members and ELF2∆ truncation mutant in HeLa cells: GFP expression confirms transduction efficiency; HA staining confirms ELF family protein overexpression; DAPI confirms DNA staining; scale bar = 50 μm. (PDF 3489 kb)

Additional file 8: Figure S4. ELF subfamily protein expression. A) Gating strategy for FACS enrichment of ELF protein-expressing HeLa cells indicating total GFP^+^ population or low, medium or high GFP-expressing cells. Total CFSE-labelled GFP^+^ HeLa cells B) and low and medium GFP subpopulations C) were incubated ± dox for 3 d. D) Gating strategy of BrdU and 7-AAD staining of ELF overexpressing HeLa cells for cell cycle analysis. E) Representative differential interference microscopy (DIC) and fluorescence images of cells overexpressing ELF subfamily members. Morphologically dead or dying cells are indicated with red arrows; scale bar = 50 μm. B). (PDF 17858 kb)

Additional file 9: Table S5. Summary of validated ELF2 targets involved in B and T cell development. All targets have been validated by reporter gene assay or by EMSA. (DOC 52 kb)

Additional file 10: Figure S5. Reconstitution efficiency in ELF2^+^ retrogenic mice. A) Murine stem cell virus-based (MSCV) retroviral vector (pMIG) used for expressing HA-tagged Elf2 isoforms; primer sequences used for detecting specific isoform expression are indicated (arrowheads); a common 5’ primer within the HA-tag and 3’ primer able to detect all Elf2 isoforms were used. B) RT-qPCR of ectopic Elf2a isoform expression in the spleens of retrogenic mice after 3 months reconstitution. Analysis of GFP expression after 4 weeks in peripheral blood mononuclear cells: total C); T cell population D); B cell population E); and granulocytes F). Reconstitution efficiency in the haemopoietic compartment after 3 months. Data represents the mean ± SEM of 3 experiments each performed with 4–5 mice per experimental arm. Statistical analysis performed using Student’s *t* test (ns, not significant; *, *p* < 0.05; **, *p* < 0.01) (PDF 1218 kb)

Additional file 11: Figure S6. Analysis of lymphocytic subsets in ELF2 retrogenic mice. A) Detection of TCRβ surface expression in thymocytes. Analysis of splenic T cells for TCRβ B) and CD4 and CD8 expression C). Analysis of mature T subsets in the spleen: CD4^+^ D) or CD8^+^ E) and CD4^+^ Tregs. Data represents the mean ± SEM. of 3 experiments each performed with 4–5 mice per experimental arm. Statistical analysis performed using Student’s *t* test (ns, not significant; *, *p* < 0.05; **, *p* < 0.01). (PDF 287 kb)

Additional file 12: Figure S7. ELF2 isoform expression decreases during ATRA-induced myeloid differentiation. A) MPRO cells were induced to differentiate with 10 μM all-trans retinoic acid (ATRA) and were co-stained with FITC-conjugated anti-Gr-1 antibodies and propidium iodide (PI) DNA dye. Stained MPRO cells were FACS-enriched for different Gr-1 populations: Gr-1^Neg^, Gr-1^Low^, Gr-1^Mid^, and Gr-1^High^. B) May-Grünwald-Giemsa staining of treated MPRO cells: Gr-1^Neg^ cells showing predominantly promyelocytes (line-arrows); Gr-1^Low^ cells showing promyelocytes and myelocytes (closed arrows); Gr-1^Mid^ cells showing myelocytes and granulocytes (open arrows); and Gr-1^High^ showing mature granulocytes. Scale bars represent 25 μm. C) Each population was examined by RT-qPCR to measure marker genes differentially expressed during granulopoiesis, including cathepsin G (*Ctsg*), lactoferrin (*Ltf*) and metalloproteinase 9 (*Mmp9*). Gene expression was normalised to β-actin and expressed relative to the Gr-1^Neg^ population (set as 1.0). Error bars represent SEM from 4 independent replicates, each performed in duplicate. D) Expression of *Elf2* isoforms was examined as in C). Two-sided *t* test was performed to compare Gr-1^High^ to Gr-1^Neg^ for each *Elf2* isoform (*p* < 0.01**, *p* < 0.001 ***) (PDF 151 kb)
