# Supplementary material for: The antiproliferative ELF2 isoform, ELF2B, induces apoptosis in vitro and perturbs early lymphocytic development in vivo
Source: J Hematol Oncol. 2017 Mar 28;10:75. doi: 10.1186/s13045-017-0446-7 (PMC5371273; doi:10.1186/s13045-017-0446-7)
Supplement: Supplementary file 1 — PCR primers used in this study (DOC 69 kb) [file 13045_2017_446_MOESM1_ESM.doc]

Supplementary Table 1: PCR primers used in this study.

| **Target** | **Name** | **Sequence** | **Purpose** |
| --- | --- | --- | --- |
| ***ELF2A1, ELF2A2*** | ELF2-Af | GGAGAATTCAGAAACAGTGGAAGC | Distinguish mouse *ELF2* isoforms |
| ***ELF2B1, ELF2B2*** | ELF2-Bf | TAAACATGGCGACGTCTCTGC | Distinguish mouse *ELF2* isoforms |
| ***ELF2A1, ELF2B1*** | ELF2-ABr1 | GCATGGATAAACTCAGGACTTCTTG | Distinguish mouse *ELF2* isoforms |
| ***ELF2A2, ELF2B2*** | ELF2-ABr2 | TCTGGAGTTGATATACAAGGAGGG | Distinguish mouse *ELF2* isoforms |
| ***HA-tagged Elf2*** | BMT-for | ATCCTTATGACGTGCCTGAC | Detection of HA-tagged mouse ELF2 |
|  | BMT-rev | ACAGTGAGCATTACTGCTG |  |
| ***β-actin*** | b-actin f(m) | CGTGGGCCGCCCTAGGCACCA | qRT-PCR for *β-actin* (mouse) |
|  | b-actin r(m) | TTGGCCTTAGGGTTCAGGGGG |  |
| ***β2m*** | mB2M-RT-5’ | CATGGCTCGCTCGGTGACC | qRT-PCR for *β2m* (mouse) |
|  | mB2M-RT-3’ | AATGTGAGGCGGGTGGAACTG |  |
| ***Gapdh*** | GAPDH-87(F) | TGCACACCAACTGCTTAGC | qRT-PCR for *Gapdh* (mouse) |
|  | GAPDH-87(R) | GGCATGGACTGTGGTCATGAG |  |
| ***Hprt*** | mHPRT-qPCR-5’ | AGTGTTGGATACAGGCCAGAC | qRT-PCR for *Hprt* (mouse) |
|  | mHPRT-qPCR-3’ | CGTGATTCAAATCCCTGAAGT |  |
| ***Tbp*** | mTBP-RT-F | CTTCGTGCAAGAAATGCTGAAT | qRT-PCR for *Tbp* (mouse) |
|  | mTBP-RT-R | CAGTTGTCCGTGGCTCTCTTATT |  |
| ***Ywhaz*** | mYWHAZ-RT-F | GCTGGTGATGACAAGAAAGGAATT | qRT-PCR for *Ywhaz* (mouse) |
|  | mYWHAZ-RT-R | GGTGTGTCGGCTGCATCTC |  |
| ***ctsg*** | mCtpsG-F | GAGTCCAGAAGGGCTGAGTG | qRT-PCR for *Ctsg* (mouse) |
|  | mCtpsG-R | CCTTTCTCGCATTTGGATGT |  |
| ***ltf*** | mLtf-F | AAACAAGCATCGGGATTCCAG | qRT-PCR for *Ltf* (mouse) |
|  | mLtf-R | ACAATGCAGTCTTCCGTGGTG |  |
| ***mmp9*** | mMMP9-F | CATTCGCGTGGATAAGGAGT | qRT-PCR for *Mmp9* (mouse) |
|  | mMMP9-R | TCACACGCCAGAAGAATTTG |  |
| ***ELF2A*** | ELF2A_P1_(-0.5)-ChIP-5’ | AGTCCTGAGTTGGGGATGC | ChIP of *ELF2A* P_1_ 500 bp upstream of TSS |
|  | ELF2A_P1_(-0.5)-ChIP-3’ | AAGACGCTCGTCACTTGTCC |  |
| ***ELF2A*** | ELF2A_P1_TSS-ChIP-5’ | GGAGGGGAGGAGCACTCTG | ChIP of *ELF2A* P_1_ proximal to TSS |
|  | ELF2A_P1_TSS-ChIP-3’ | CTCCACAGGGAGAGAAGGAG |  |
| ***ELF2A*** | ELF2A_P1_(+0.5)-ChIP-5’ | AGCGGGACTTGGAGAAAGG | ChIP of *ELF2A* P_1_ 500 bp downstream of TSS |
|  | ELF2A_P1_(+0.5)-ChIP-3’ | CAGTTCAGTCCGCTTCTCG |  |
| ***ELF2A*** | ELF2A_P2_TSS-ChIP-5’ | GTGCGACATATGGCTTTGG | ChIP of *ELF2A* P_2_ proximal to TSS |
|  | ELF2A_P2_TSS-ChIP-3’ | CACAGTCCCATCATACTATCACC |  |
| ***ELF2B*** | hELF2b(-750)-ChIP-5' | TCGGATCATAAAAATTGTCACC | ChIP of human *ELF2B* promoter (P_3_) 750 bp upstream of TSS |
|  | hELF2b(-750)-ChIP-3' | TTTCTGTTTGGCTCACTTGG |  |
| ***ELF2B*** | hELF2b(TSS)-ChIP-5' | GTGGTGGGGTTTCTCAGC | ChIP of human *ELF2B* promoter (P_3_) proximal to TSS |
|  | hELF2b(TSS)-ChIP-3' | CCTCCATTACTTCATTCACTCC |  |
| ***ELF2B*** | hELF2b(+1.5)-ChIP-5' | CAAACCTCCCACCACAGC | ChIP of human *ELF2B* promoter (P_3_) 1.5 kb downstream of TSS |
|  | hELF2b(+1.5)-ChIP-3' | CTTAAGTCCGTGCCCTTCC |  |
| ***VCP*** | hVCP-ChIP-5' | CTTCTCAACGCTGCCTCCAAC | ChIP of human *VCP* proximal to TSS |
|  | hVCP-ChIP-3' | GGTGAAAGGTGAATTGAGTCAGCA |  |
| ***PYGO2*** | hPygo2-ChIP-5’ | AGGCGTAGCGTCTCGTCCG | ChIP of human *PYGO2* proximal to TSS |
|  | hPygo2-ChIP-3’ | GAGCTGCAGCAACCACAAAGTG |  |
| ***LYN*** | hLyn-ChIP-5' | CTTCCGTCCGTCCCTACA | ChIP of human *LYN* proximal to TSS |
|  | hLyn-ChIP-3' | GAGGAACTGGCTGTCTCTGG |  |
| ***PLAU*** | hPLAU-ChIP-5’ | CACGCTTCATAACGGTCTCC | ChIP of human *PLAU* intergenic region |
|  | hPLAU-ChIP-3’ | AGAGCCAACCTTGCTACTTCC |  |
| ***LMO2*** | hLMO2-ChIP-5’ | TGATGCTGCTGTTTGTATTGC | ChIP of human *LMO2* proximal promoter adjacent to TSS |
|  | hLMO2-ChIP-5’ | GACCGTGCGTCTCTCTCC |  |
| ***H19*** | H19-ex4/5-ChIP-5' | TCATCCCGCTGGAGGAGCTCAGCT | Control ChIP from *H19* exon 4 and 5 |
|  | H19-ex4/5-ChIP-3' | TGCTGCACTTTACAACCACTGCAC |  |
