# Supplementary material for: The antiproliferative ELF2 isoform, ELF2B, induces apoptosis in vitro and perturbs early lymphocytic development in vivo
Source: J Hematol Oncol. 2017 Mar 28;10:75. doi: 10.1186/s13045-017-0446-7 (PMC5371273; doi:10.1186/s13045-017-0446-7)
Supplement: Supplementary file 2 — List of primary and secondary antibodies used in immunofluorescence (IF) and western blot (WB) analysis. All antibodies were diluted to their working concentrations in the appropriate blocking solution (DOC 43 kb) [file 13045_2017_446_MOESM2_ESM.doc]

Supplementary Table 2. List of primary and secondary antibodies used in immunofluorescence (IF) and Western blot (WB) analysis. All antibodies were diluted to their working concentrations in the appropriate blocking solution.

|  | Specificity | Type | Species | Conjugate | Working Dilution | Blocking Solution | Source |
| --- | --- | --- | --- | --- | --- | --- | --- |
| 1° antibody | ELF2A | Polyclonal | Rabbit | - | WB: 1:1000 | 5% skim milk | IMVS; Australia |
|  |  |  |  |  | IF: 1:50 | 20% BlokHen |  |
|  | ELF2B | Polyclonal | Rabbit | - | WB: 1:1000 | 3% BSA + 2% skim milk | IMVS; Australia |
|  |  |  |  |  | IF: 1:50 | 20% BlokHen |  |
|  | ELF1 | Polyclonal | Rabbit | - | WB 1:1000 | 3% BSA + 2% skim milk | Bethyl Laboratories  A301-443A |
|  | ELF4 | Polyclonal | Rabbit | - | WB 1:1000 | 3% BSA + 2% skim milk | Bethyl Laboratories  A302-659A |
|  | VCP | Polyclonal | Rabbit | - | WB: 1:1000 | 5% skim milk | Cell Signaling  2648S |
|  | HA | Monoclonal | Mouse | - | WB: 1:1000 | 5% skim milk | Covance |
|  |  |  |  |  | IF: 1:100 | 20% BlokHen |  |
|  | α-Tubulin | Monoclonal | Mouse | - | WB: 1:5000 | 5% skim milk | Santa Cruz |
|  | GAPDH | Monoclonal | Mouse | - | WB: 1:5000 | 5% skim milk | Abcam |
|  | Lmnb1 | Monoclonal | Rabbit | - | WB: 1:2500 | 5% skim milk | Abcam |
| 2° antibody | Rabbit IgG | Polyclonal | Donkey | HRP | WB: 1:5000 | 5% skim milk | Chemicon |
|  | Mouse IgG | Polyclonal | Donkey | HRP | WB: 1:5000 | 5% skim milk | Chemicon |
|  | Rabbit IgG | Polyclonal | Goat | Alexa 594 | IF: 1:500 | 20% BlokHen | Invitrogen |
|  | Mouse IgG | Polyclonal | Goat | Alexa 594 | IF: 1:500 | 20% BlokHen | Invitrogen |
