# Supplementary material for: The antiproliferative ELF2 isoform, ELF2B, induces apoptosis in vitro and perturbs early lymphocytic development in vivo
Source: J Hematol Oncol. 2017 Mar 28;10:75. doi: 10.1186/s13045-017-0446-7 (PMC5371273; doi:10.1186/s13045-017-0446-7)
Supplement: Supplementary file 3 — Antibody-fluorophore conjugates and filter combinations used to distinguish haemopoietic-specific cell surface markers (DOC 40 kb) [file 13045_2017_446_MOESM3_ESM.doc]

**Supplementary Table 3 Antibody-fluorophore conjugates and filter combinations used to distinguish haemopoietic-specific cell surface markers.**

| **Tissue** | **Populations** | **Laser and Bandpass filter** | | | | | | |
| --- | --- | --- | --- | --- | --- | --- | --- | --- |
|  |  | **B530** | **B710** | **R670** | **R780** | **V450** | **YG582** | **YG670** |
| **Thymus** | **T cells:**  **DN, DP, CD4, CD8** | GFP | CD4-PerCPCy5.5 | TCRβ-APC |  |  | CD8-PE |  |
| **Thymus** | **T cells:**  **DN1, DN2, DN3, DN4** | GFP |  | CD44-Alexa647 | CD25-APC/Cy7 | CD4/CD8-biotin +Strept-V450 |  |  |
| **Spleen** | **B cells, Granulocytes, Dendritic cells** | GFP |  | CD11c-APC |  | Gr1-V450 | B220-PE | CD11b-PECy5 |
| **Spleen** | **T cells:**  **CD4, CD8** | GFP | CD4-PerCPCy5.5 | TCRβ-APC |  |  | CD8-PE |  |
| **Spleen** | **T cells:  CD4 (Naïve; Memory) CD8 (Naïve; Memory)** | GFP | CD4-PerCPCy5.5 | CD44-Alexa647 | CD62L- APC/Cy7 |  | CD8-PE |  |
| **Spleen** | **Regulatory T cells** | GFP | CD4-PerCPCy5.5 |  | CD25-APC/Cy7 |  | CD8-PE |  |
| **Spleen** | **B cells: Immature; Follicular; Marginal** | GFP | B220-PerCPCy5.5 | CD21-APC | IgM-Biotin APC/Cy7 | IgD-V450 (Pacific Blue) | CD23-PE |  |
| **Peritoneal Cavity** | **B1 B cells** | GFP | CD5-PerCPCy5.5 | CD11b-APC | IgM-Biotin APC/Cy7 | IgD-V450 | B220-PE |  |
| **Bone Marrow** | **B cells, Granulocytes, Dendritic cells** | GFP |  | CD11c-APC |  | Gr-1 V450 | B220-PE | CD11b-PECy5 |
| **Bone Marrow** | **B cells:**  **Pre B; Immature B; Recirculating B** | GFP |  | B220-APC |  | IgM-Biotin V450 | CD43-PE |  |
| **Bone Marrow** | **Haemopoietic stem cells (LSK)** | GFP |  | Sca-1-APC |  | Lin-Negative (Biotin) Gr-1; B220; Ter119; CD11b; TCRb | c-kit- PE |  |
