# Supplementary figures and images for: The antiproliferative ELF2 isoform, ELF2B, induces apoptosis in vitro and perturbs early lymphocytic development in vivo
Source: J Hematol Oncol. 2017 Mar 28;10:75. doi: 10.1186/s13045-017-0446-7 (PMC5371273; doi:10.1186/s13045-017-0446-7)

Supplementary Figure 1

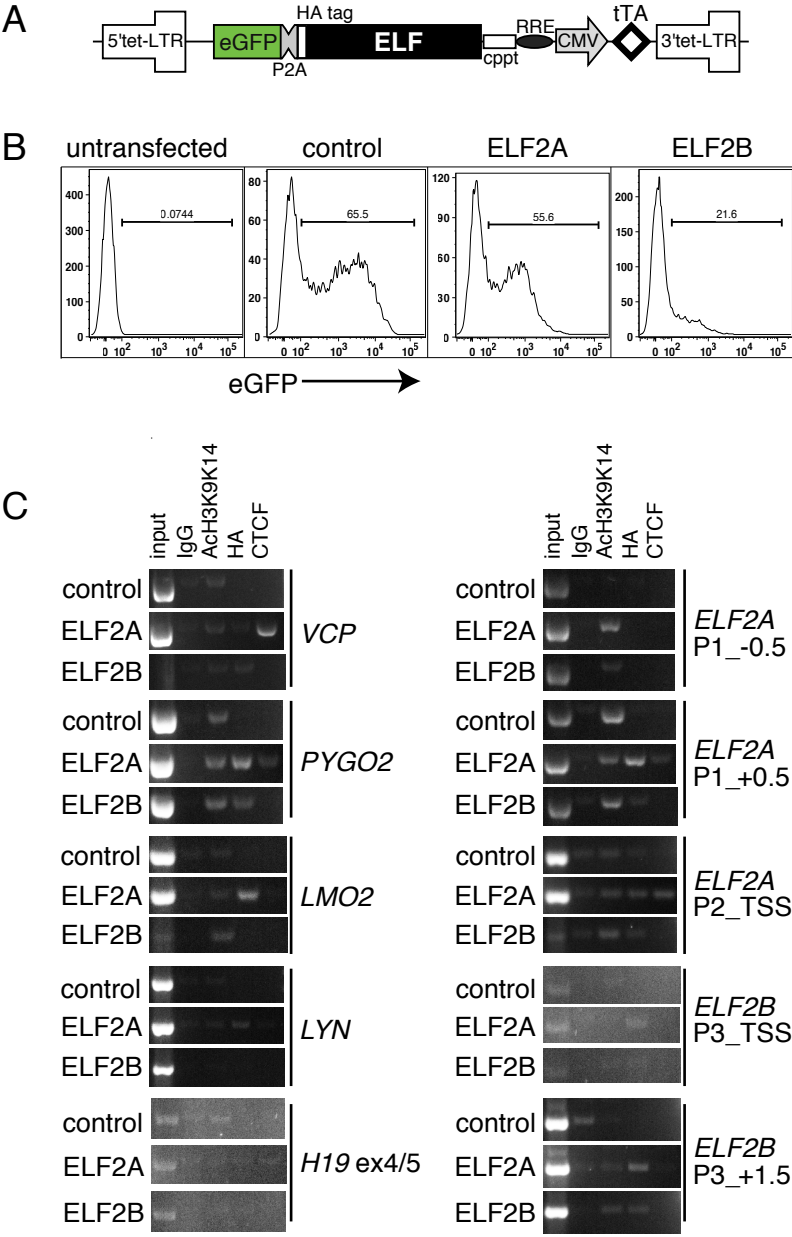

Supplement: Supplementary file 4 — Confirmation of DNA binding of ELF2 isoforms by ChIP. A) The doxycycline-regulatable ‘dox-off’ lentiviral vector used to co-express eGFP and ELF2 isoforms. B) Flow cytometric analysis of HEK293T cells transfected with eGFP only (control)-, HA-ELF2A- and HA-ELF2B-containing vectors. C) ChIP PCR of known ELF2 targets (VCP, PYGO2, LMO2, and LYN promoters), novel ELF2-binding sites in ELF2 promoter regions (P1, P2, and P3) as well as a negative control region spanning H19 exons 4 and 5. (PDF 1124 kb) [file 13045_2017_446_MOESM4_ESM.pdf]

Supplementary Figure 2

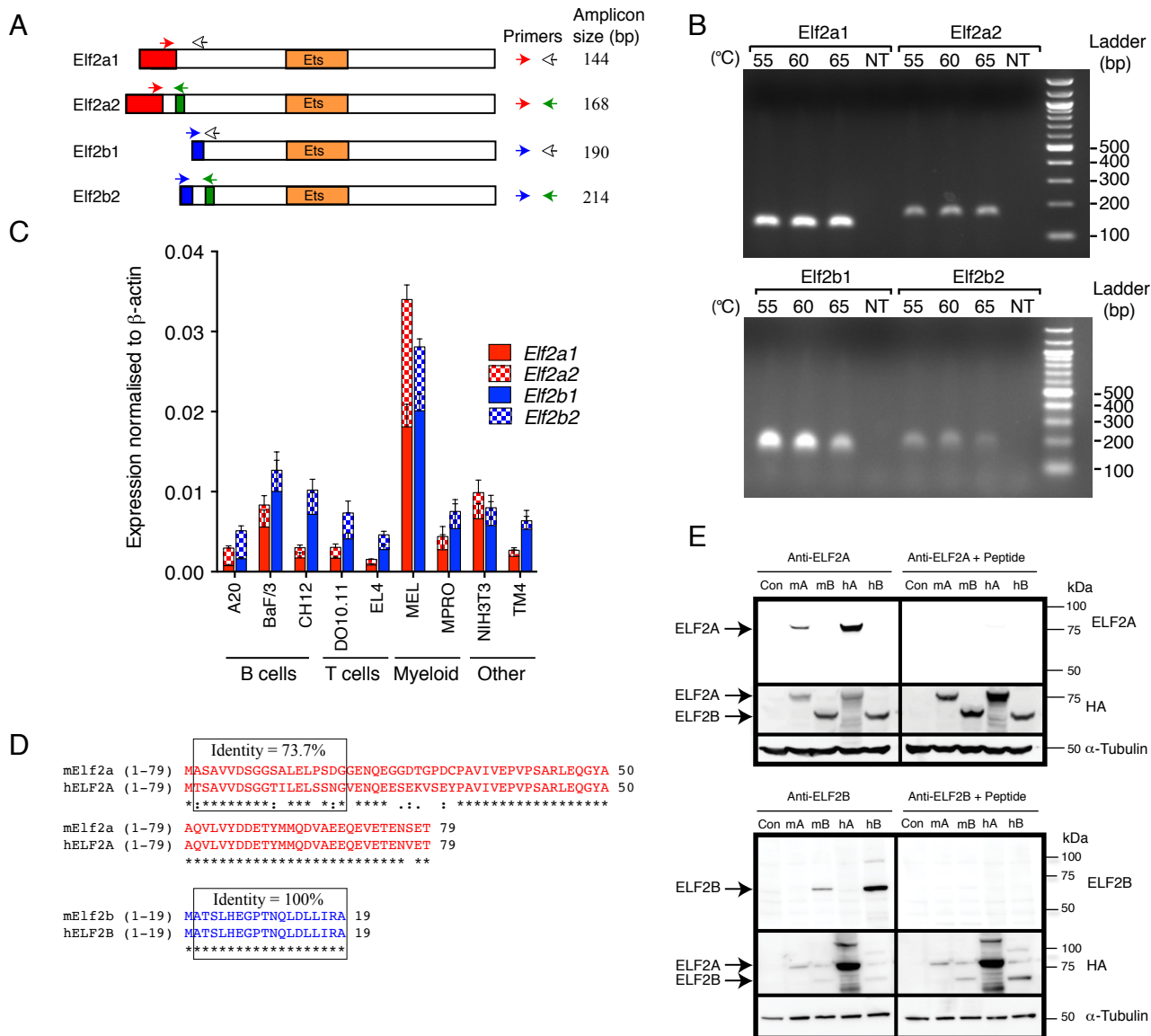

Supplement: Supplementary file 5 — Validation of reagents used to detect ELF2 isoform expression. Design A) and validation B) of RT-qPCR primers used to detect Elf2a and Elf2b major and minor isoforms with expected amplicon sizes (bp). C) RT-qPCR detection of Elf2 isoform expression in murine haemopoietic cell lines. D) Specific N-terminal sequences used as immunising peptides to produce isoform-specific antibodies. The amino acid identity between mouse and human sequences is shown. E) Validation of specificity and species cross-reactivity of ELF2A and ELF2B antibodies in control-transduced (GFP vector only; Con) HEK293T cells and cells transduced with mouse Elf2A (mA), mouse Elf2b (mB), human ELF2A (hA), or human ELF2B (hB)-containing lentiviral vectors (PDF 1535 kb) [file 13045_2017_446_MOESM5_ESM.pdf]

Supplementary Figure 3

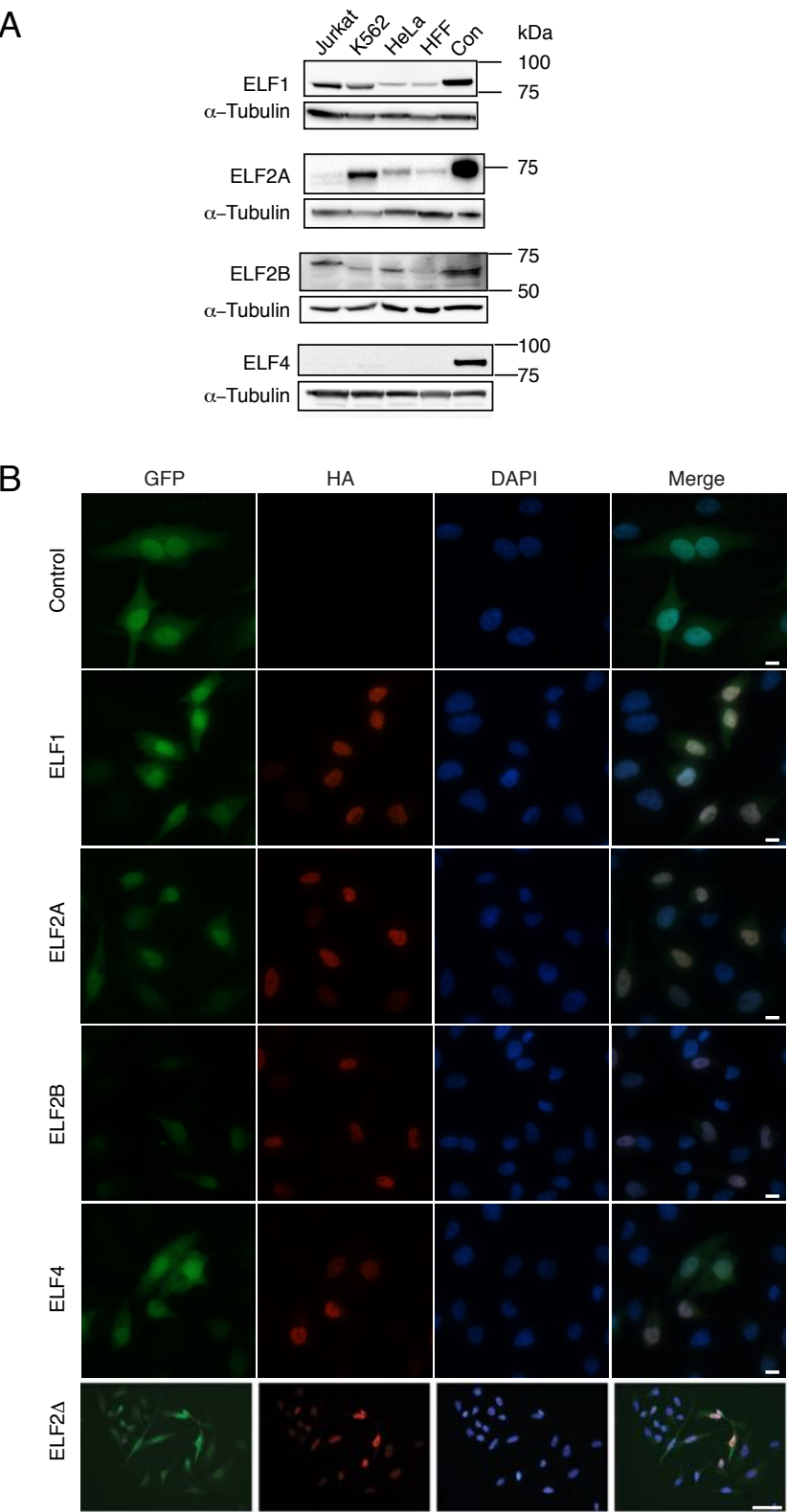

Supplement: Supplementary file 7 — Confirmation of ELF protein expression in vitro. A) Determination of endogenous ELF family protein levels in immortalised and primary cells; Con = HeLa cells overexpressing the respective HA-tagged ELF protein. Numbers indicate molecular weight markers (in kDa). B) Confirmation of subcellular localisation of ELF family members and ELF2∆ truncation mutant in HeLa cells: GFP expression confirms transduction efficiency; HA staining confirms ELF family protein overexpression; DAPI confirms DNA staining; scale bar = 50 μm. (PDF 3489 kb) [file 13045_2017_446_MOESM7_ESM.pdf]

Supplementary Figure 4

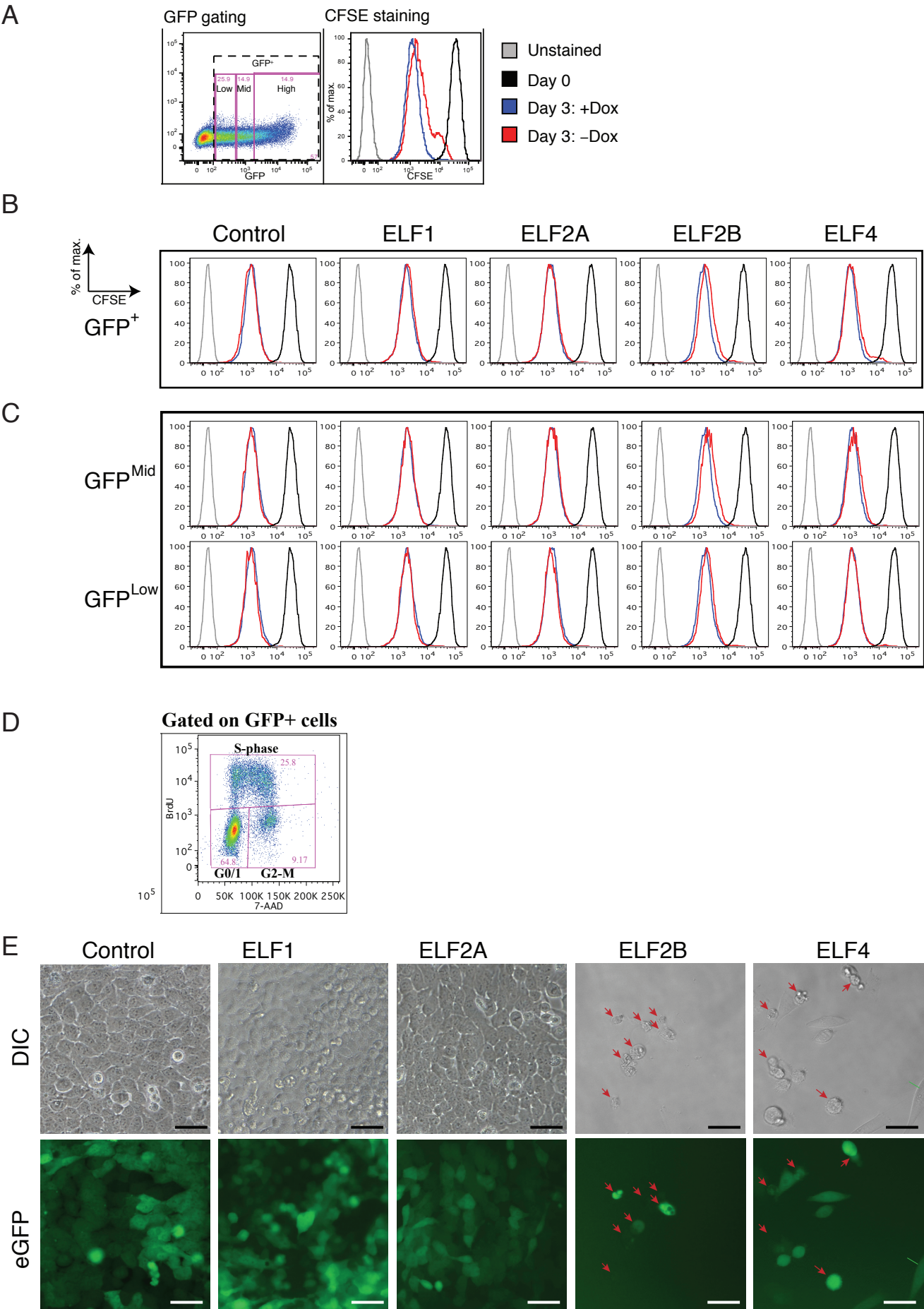

Supplement: Supplementary file 8 — ELF subfamily protein expression. A) Gating strategy for FACS enrichment of ELF protein-expressing HeLa cells indicating total GFP+ population or low, medium or high GFP-expressing cells. Total CFSE-labelled GFP+ HeLa cells B) and low and medium GFP subpopulations C) were incubated ± dox for 3 d. D) Gating strategy of BrdU and 7-AAD staining of ELF overexpressing HeLa cells for cell cycle analysis. E) Representative differential interference microscopy (DIC) and fluorescence images of cells overexpressing ELF subfamily members. Morphologically dead or dying cells are indicated with red arrows; scale bar = 50 μm. B). (PDF 17858 kb) [file 13045_2017_446_MOESM8_ESM.pdf]

Supplementary Figure 5

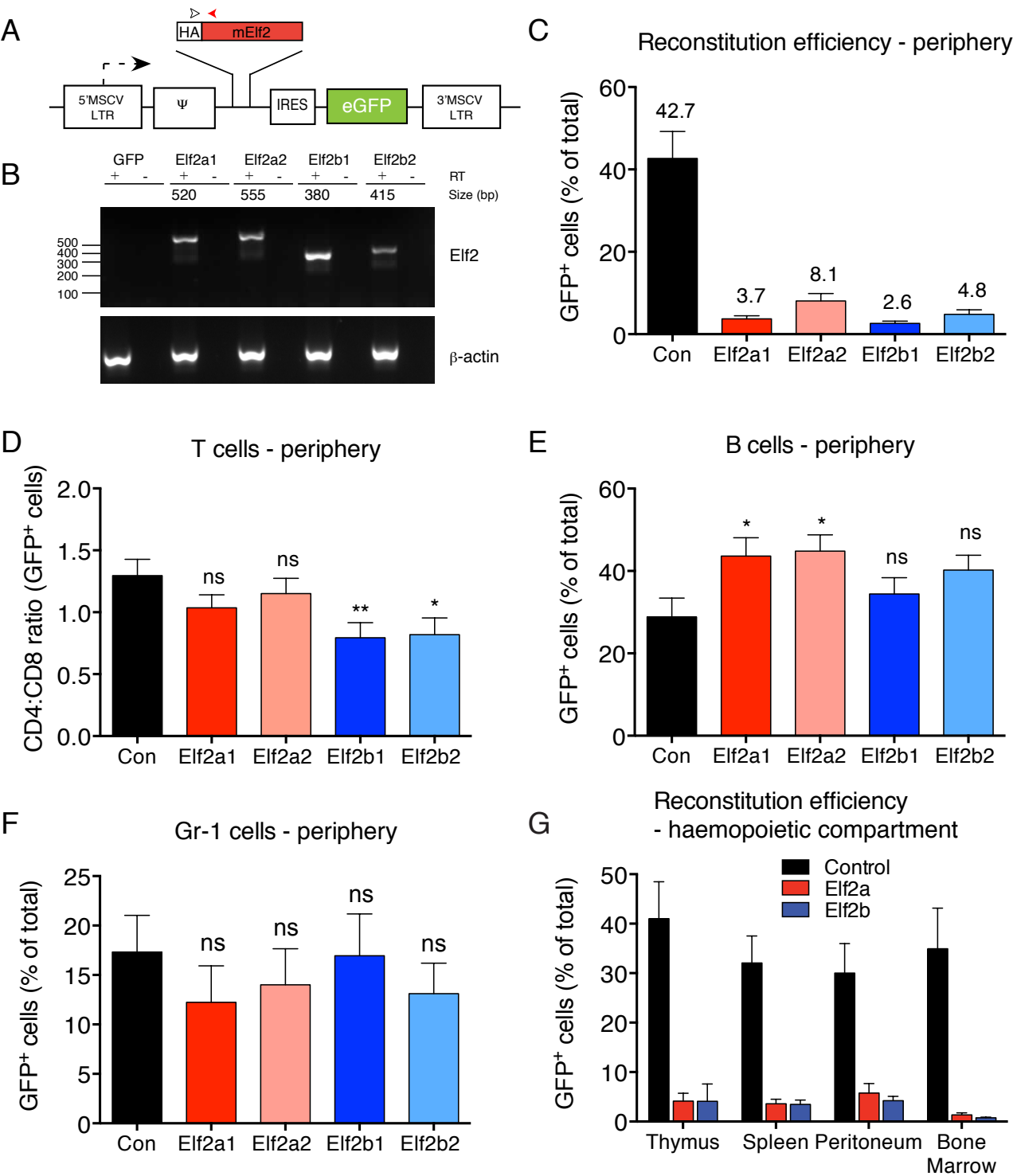

Supplement: Supplementary file 10 — Reconstitution efficiency in ELF2+ retrogenic mice. A) Murine stem cell virus-based (MSCV) retroviral vector (pMIG) used for expressing HA-tagged Elf2 isoforms; primer sequences used for detecting specific isoform expression are indicated (arrowheads); a common 5’ primer within the HA-tag and 3’ primer able to detect all Elf2 isoforms were used. B) RT-qPCR of ectopic Elf2a isoform expression in the spleens of retrogenic mice after 3 months reconstitution. Analysis of GFP expression after 4 weeks in peripheral blood mononuclear cells: total C); T cell population D); B cell population E); and granulocytes F). Reconstitution efficiency in the haemopoietic compartment after 3 months. Data represents the mean ± SEM of 3 experiments each performed with 4–5 mice per experimental arm. Statistical analysis performed using Student’s t test (ns, not significant; *, p < 0.05; **, p < 0.01) (PDF 1218 kb) [file 13045_2017_446_MOESM10_ESM.pdf]

Supplementary Figure 6

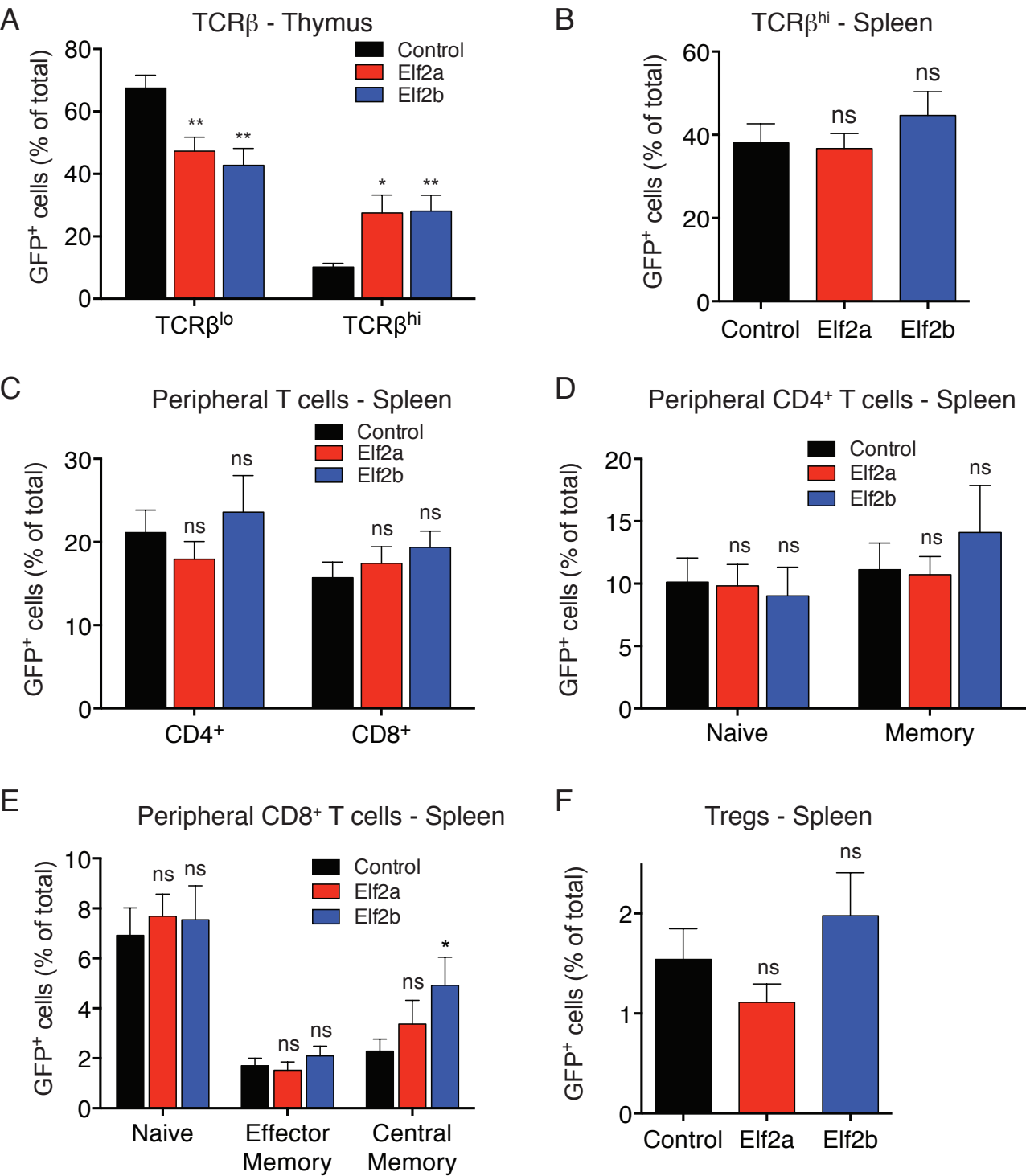

Supplement: Supplementary file 11 — Analysis of lymphocytic subsets in ELF2 retrogenic mice. A) Detection of TCRβ surface expression in thymocytes. Analysis of splenic T cells for TCRβ B) and CD4 and CD8 expression C). Analysis of mature T subsets in the spleen: CD4+ D) or CD8+ E) and CD4+ Tregs. Data represents the mean ± SEM. of 3 experiments each performed with 4–5 mice per experimental arm. Statistical analysis performed using Student’s t test (ns, not significant; *, p < 0.05; **, p < 0.01). (PDF 287 kb) [file 13045_2017_446_MOESM11_ESM.pdf]

Supplementary Figure 7

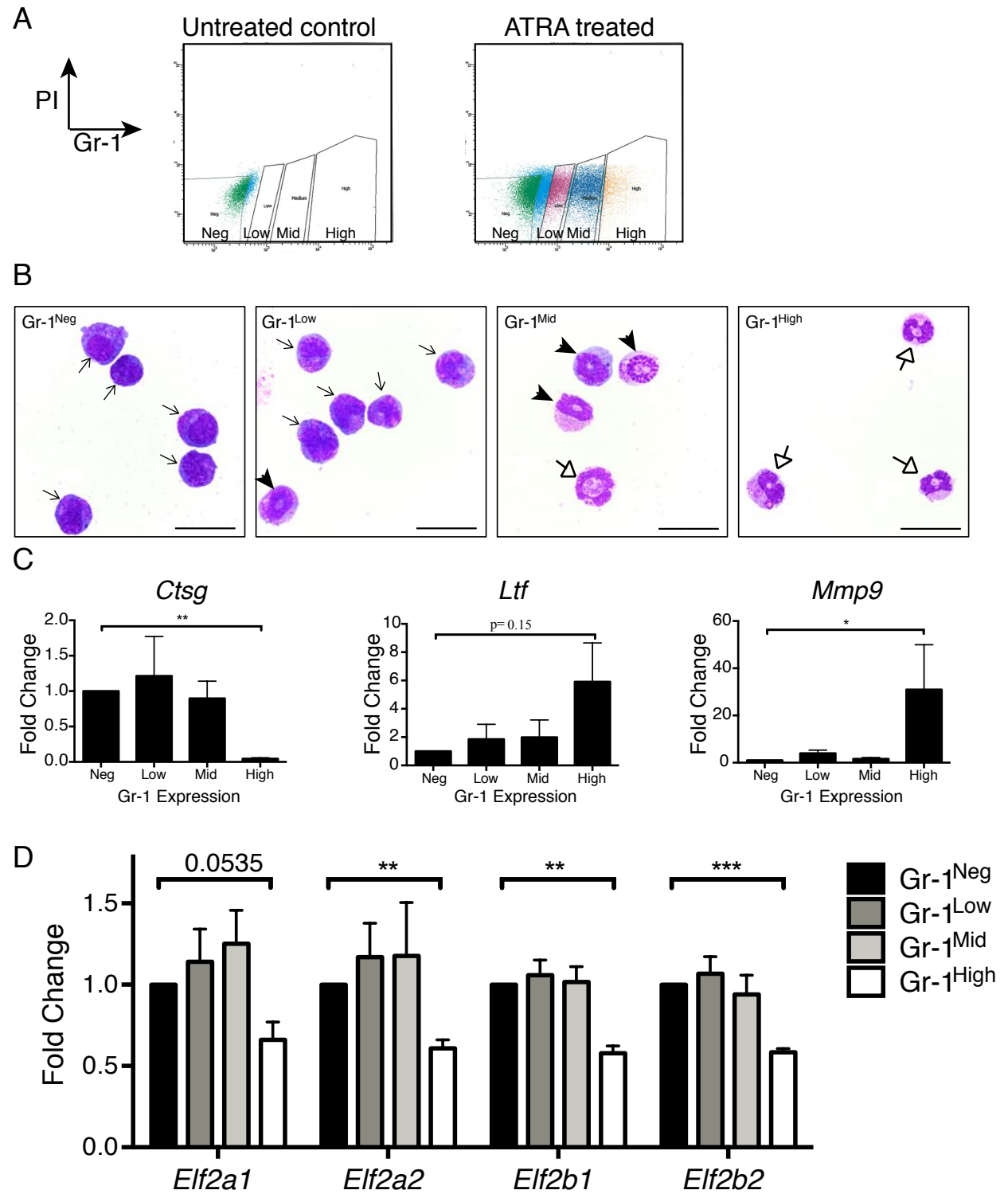

Supplement: Supplementary file 12 — ELF2 isoform expression decreases during ATRA-induced myeloid differentiation. A) MPRO cells were induced to differentiate with 10 μM all-trans retinoic acid (ATRA) and were co-stained with FITC-conjugated anti-Gr-1 antibodies and propidium iodide (PI) DNA dye. Stained MPRO cells were FACS-enriched for different Gr-1 populations: Gr-1Neg, Gr-1Low, Gr-1Mid, and Gr-1High. B) May-Grünwald-Giemsa staining of treated MPRO cells: Gr-1Neg cells showing predominantly promyelocytes (line-arrows); Gr-1Low cells showing promyelocytes and myelocytes (closed arrows); Gr-1Mid cells showing myelocytes and granulocytes (open arrows); and Gr-1High showing mature granulocytes. Scale bars represent 25 μm. C) Each population was examined by RT-qPCR to measure marker genes differentially expressed during granulopoiesis, including cathepsin G (Ctsg), lactoferrin (Ltf) and metalloproteinase 9 (Mmp9). Gene expression was normalised to β-actin and expressed relative to the Gr-1Neg population (set as 1.0). Error bars represent SEM from 4 independent replicates, each performed in duplicate. D) Expression of Elf2 isoforms was examined as in C). Two-sided t test was performed to compare Gr-1High to Gr-1Neg for each Elf2 isoform (p < 0.01**, p < 0.001 ***) (PDF 151 kb) [file 13045_2017_446_MOESM12_ESM.pdf]
