# Supplementary material for: The antiproliferative ELF2 isoform, ELF2B, induces apoptosis in vitro and perturbs early lymphocytic development in vivo
Source: J Hematol Oncol. 2017 Mar 28;10:75. doi: 10.1186/s13045-017-0446-7 (PMC5371273; doi:10.1186/s13045-017-0446-7)
Supplement: Supplementary file 6 — Somatic mutations in ELF2 in cancer. Mutations are compiled from the TCGA CBIO portal and COSMIC databases. Mutations for ELF2A are shown; no mutations in ELF2B’s 19 aa N-terminus have been recorded (DOC 99 kb) [file 13045_2017_446_MOESM6_ESM.doc]

**Supplementary Table 4** Somatic mutations in ELF2 in cancer. Mutations are compiled from the TCGA CBIO portal and COSMIC databases. Mutations for ELF2A are shown; no mutations in ELF2B’s 19 aa N-terminus have been recorded.

ELF2A

| **Position** | **Sample ID** | **Cancer Study** | **Mutation** | **Type** |
| --- | --- | --- | --- | --- |
| 1 | TCGA-D3-A3C8-06 | Melanoma (TCGA) | M1I | Non-start |
| 1 | TCGA-B5-A11N-01 | Uterine (TCGA pub) | M1I | Non-start |
| 11 | DU_145 | NCI-60 | T11A | Missense |
| 20 | 134421 | Lung SC (JHU) | V20I | Missense |
| 22 | TCGA-BS-A0UF-01 | Uterine (TCGA pub) | N22H | Missense |
| 40 | 4138050 | Ovary (COSMIC) | V40G | Missense |
| 54 | YUKIL | Melanoma (Yale) | L54P | Missense |
| 64 | ccRCC_3 | ccRCC (U Tokyo) | Q64E | Missense |
| 65 | SK_MEL_2 | NCI-60 | D65N | Missense |
| 67 | 4680740 | Large intestine (COSMIC) | A67T | Missense |
| 70 | 480889 | Kidney (COSMIC) | Q70E | Missense |
| 74 | TCGA-XE-AAOJ-01 | Testicular germ cell (TCGA) | T74N | Missense |
| 83 | 5344619 | Large intestine (COSMIC) | S83* | Nonsense |
| 83 | TCGA-HU-A4GP-01 | Stomach (TCGA pub) | S83L | Missense |
| 99 | 480888 | Kidney (COSMIC) | A99V | Missense |
| 111 | 4882674 | Oesophagus (COSMIC) | L111F | Missense |
| 123 | 3011138 | Large intestine (COSMIC) | A123D | Missense |
| 125 | 5500746 | Biliary Tract (COSMIC) | R125S | Missense |
| 134 | 05-123E2_LN | Prostate (FHCRC) | V134del | In frame deletion |
| 136 | 310855 | Melanoma (COSMIC) | V136L | Missense |
| 136 | S00837 | Lung SC (CLCGP) | V136L | Missense |
| 152 | TCGA-AG-A002-01 | Colorectal (TCGA pub) | E152* | Nonsense |
| 180 | 3917185 | Melanoma (COSMIC) | P180S | Missense |
| 188 | TCGA-FW-A3R5-06 | Melanoma (TCGA) | P188R | Missense |
| 194 | 1051361 | Endometrium (COSMIC) | N194H | Missense |
| 194 | TCGA-BS-A0UF-01 | Uterine (TCGA pub) | N194H | Missense |
| 195 | 4787759 | Liver (COSMIC) | T195K | Missense |
| 188 | TCGA-44-2656-01 | Lung adeno (TCGA pub) | P188R | Missense |
| 215 | 109747 | Melanoma (COSMIC) | Y215F | Missense |
| 217 | pfg125T | Stomach (Pfizer UHK) | K217Q | Missense |
| 217 | TCGA-EE-A2A2-06 | Melanoma (TCGA) | K217E | Missense |
| 219 | TCGA-BW-A5NQ-01 | Liver (TCGA) | T219N | Missense |
| 222 | TCGA-B5-A0JY-01 | Uterine (TCGA) | E222D | Missense |
| 238 | TCGA-BP-4968-01 | ccRCC (TCGA pub) | W238* | Nonsense |
| 241 | 4680738 | Large intestine (COSMIC) | H241fs*2 | Frameshift |
| 244 | 5466056 | Large intestine (COSMIC) | K244T | Missense |
| 250 | TCGA-55-7574-01 | Lung adeno (TCGA pub) | E250K | Missense |
| 251 | TCGA-AX-A0J1-01 | Uterine (TCGA pub) | T251I | Missense |
| 274 | TCGA-AA-3672-01 | Colorectal (TCGA pub) | V274A | Missense |
| 296 | 3781756 | Pancreas (Cosmic) | N296S | Missense |
| 313 | 4680736 | Large intestine (COSMIC) | L313P | Missense |
| 325 | TCGA-05-4432-01 | Lung adeno (TCGA pub) | V325F | Missense |
| 330 | TCGA-D9-A6EC-06 | Melanoma (TCGA) | N330H | Missense |
| 348 | ccRCC_18 | ccRCC (U Tokyo) | N348H | Missense |
| 351 | MM-0244 | MM (Broad) | S351P | Missense |
| 360 | MSKCC-0343_R | Bladder (DFARBER_MSKCC 2014) | S360Yfs*40 | Frameshift |
| 391 | TCGA-C5-A2LZ-01 | Cervical (TCGA) | Q391* | Nonsense |
| 392 | TCGA-AP-A059-01 | Uterine (TCGA) | K392T | Missense |
| 394 | ESO-114 | Esophagus (Broad) | S394* | Nonsense |
| 400 | TCGA-EJ-A8FS-01 | Prostate (TCGA 2015) | S400* | Nonsense |
| 401 | 5459087 | Large intestine (COSMIC) | V401I | Missense |
| 403 | TCGA-EW-A1PB-01 | Breast (TCGA 2015) | A403V | Missense |
| 404 | TCGA-B5-A11E-01 | Uterine (TCGA pub) | G404A | Missense |
| 406 | 1194157 | Lung (COSMIC) | P406T | Missense |
| 409 | TCGA-B0-4823-01 | ccRCC (TCGA pub) | T409A | Missense |
| 419 | TCGA-44-2656-01 | Lung adeno (TCGA pub) | P419R | Missense |
| 446 | 587376 | Colorectal (Genentech) | K446N | Missense |
| 454 | TCGA-B3-3926-01 | pRCC (TCGA) | Q454E | Missense |
| 456 | 5443115 | Oesophagus (COSMIC) | A456V | Missense |
| 459 | 587224 | Colorectal (Genentech) | Q459R | Missense |
| 466 | TCGA-FD-A3SQ-01 | Bladder (TCGA 2014) | L466V | Missense |
| 467 | TCGA-BH-A0EE-01 | Breast (TCGA 2015) | T467S | Missense |
| 470 | 3727049 | Melanoma (COSMIC) | G470R | Missense |
| 474 | TCGA-ND-A4WC-01 | Uterine CS (TCGA) | I474S | Missense |
| 500 | 3720031 | Haemopoietic (COSMIC) | M500V | Missense |
| 508 | TCGA-44-6145-01 | Lung adeno (TCGA pub) | Q508H | Missense |
| 510 | 3011123 | Pancreas (COSMIC) | P510A | Missense |
| 511 | 4680734 | Large intestine (COSMIC) | P511L | Missense |
| 519 | TCGA-CQ-5324-01 | Head & neck (TCGA pub) | K519Rfs*18 | Frameshift |
| 532 | LUAD-RT-S01777 | Lung adeno (Broad) | Q532P | Missense |
| 544 | 4680732 | Large intestine (COSMIC) | E544G | Missense |
| 556 | 3428176 | Breast (COSMIC) | V556I | Missense |
| 556 | 3428176 | Large intestine (COSMIC) | V556I | Missense |
| 561 | TCGA-BR-4362-01 | Stomach (TCGA pub) | A561V | Missense |
| 582 | TCGA-EE-A2GC-06 | Melanoma (TCGA) | *582Eext*17 | Nonstop |
