# Supplementary material for: The antiproliferative ELF2 isoform, ELF2B, induces apoptosis in vitro and perturbs early lymphocytic development in vivo
Source: J Hematol Oncol. 2017 Mar 28;10:75. doi: 10.1186/s13045-017-0446-7 (PMC5371273; doi:10.1186/s13045-017-0446-7)
Supplement: Supplementary file 9 — Summary of validated ELF2 targets involved in B and T cell development. All targets have been validated by reporter gene assay or by EMSA. (DOC 52 kb) [file 13045_2017_446_MOESM9_ESM.doc]

**Supplementary Table 5** Summary of validated ELF2 targets involved in B and T cell development. All targets have been validated by reporter gene assay or by EMSA^1,2^.

| **Gene** | **Regulatory region** | **B** | **T** | **Function/Role** |
| --- | --- | --- | --- | --- |
| *BLK* | promoter |  |  | Blk expression starts at the late pro-B/early pre-B cell stage^3^; Crosslinking of the BCR leads to the activation of the src-family of receptor tyrosine kinases (SRK) Lyn and Blk^4^; BLK and LYN play an essential role in the pre-BCR-mediated NF-κB activation and B-cell development^5^. |
| *LYN* | promoter |  |  | Crosslinking of the BCR leads to the activation of the src-family of receptor tyrosine kinases (SRK) Lyn and Blk^4^; BLK and LYN play an essential role in the pre-BCR-mediated NF-κB activation and B-cell development^5^. |
| *IgH π* | enhancer |  |  | Immunoglobulin heavy chain enhancer π is transcriptionally active during early B-cell development^6^; ELF1 transcriptionally activates the IgH π enhancer in B cells^7^. |
| *IgH µB* | enhancer |  |  | IgH µ is transcriptionally active during pre-B-cell development, facilitating V(D)J recombination^8^. |
| *TDT* | promoter |  |  | TdT expression occurs primary lymphoid cells in the thymus and bone marrow where V(D)J recombination occurs^9^; TdT plays an essential role in the B and T-cell lymphocyte and repertoire development^10^. |
| *CD79A* | promoter |  |  | Mutations in CD79A/MB-1/Igα results in a block in B-cell development during the pro-B to pre-B cell transition^11^. |
| *JUNB* | promoter |  |  | JUNB is a negative regulator of B-cell development and proliferation^12^. JUNB plays a role in Th2 effector cell development, cytokine production and function^13^. |
| *UPA* | enhancer |  |  | Urokinase plasminogen activator is required for T cell activation and proliferation and the Th1 immune response^14^. |
| *CD79B* | promoter |  |  | Encodes Igβ, a component of the B-cell receptor signaling complex. B29/IgB is regulated by early B cell factor (EBF) in early pro-B cells^15^. |
| *IgK 3’* | enhancer |  |  | The IgK 3’ enhancer is activated in late pro-B/early pre-B cell development^16^. |
| *LCK* | promoter |  |  | LCK is a SRK involved in proximal T cell receptor signal transduction^17^. |
| *FOS* | promoter |  |  | The ubiquitous transcription factor c-FOS/AP-1 is rapidly induced upon T cell activation^18^. |
| *IL10* | promoter |  |  | IL10 is produced by innate and adaptive immune cells including lymphoid and myeloid cells^19^. |
| *CD3δ* | promoter |  |  | CD3δ and other CD3 subunits γ and ε form the scaffold allowing the assembly of the pre-TCR-CD3 complex^1^. |

**References**

1. Ji, H. B., Gupta, A., Okamoto, S., Blum, M. D., et al. T cell-specific expression of the murine CD3delta promoter. J Biol Chem 277, 47898-47906 (2002).

2. Oettgen, P., Akbarali, Y., Boltax, J., Best, J., et al. Characterization of NERF, a novel transcription factor related to the Ets factor ELF-1. Mol Cell Biol 16, 5091-5106 (1996).

3. Wasserman, R., Li, Y. S. & Hardy, R. R. Differential expression of the blk and ret tyrosine kinases during B lineage development is dependent on Ig rearrangement. J Immunol 155, 644-651 (1995).

Page 49 of 55

4. Sefton, B. M. & Taddie, J. A. Role of tyrosine kinases in lymphocyte activation. Curr Opin Immunol 6, 372-379 (1994).

5. Saijo, K., Schmedt, C., Su, I. H., Karasuyama, H., et al. Essential role of Src-family protein tyrosine kinases in NF-kappaB activation during B cell development. Nat Immunol 4, 274-279 (2003).

6. Libermann, T. A. & Baltimore, D. Pi, a pre-B-cell-specific enhancer element in the immunoglobulin heavy-chain enhancer. Mol Cell Biol 13, 5957-5969 (1993).

7. Akbarali, Y., Oettgen, P., Boltax, J. & Libermann, T. A. ELF-1 interacts with and transactivates the IgH enhancer pi site. J Biol Chem 271, 26007-26012 (1996).

8. Ong, J., Stevens, S., Roeder, R. G. & Eckhardt, L. A. 3 IgH enhancer elements shift synergistic interactions during B cell development. The Journal of Immunology 160, 4896-4903 (1998).

9. Motea, E. A. & Berdis, A. J. Terminal deoxynucleotidyl transferase: the story of a misguided DNA polymerase. Biochim Biophys Acta 1804, 1151-1166 (2010).

10. Benedict, C. L., Gilfillan, S., Thai, T. H. & Kearney, J. F. Terminal deoxynucleotidyl transferase and repertoire development. Immunol Rev 175, 150-157 (2000).

11. Minegishi, Y., Coustan-Smith, E., Rapalus, L., Ersoy, F., et al. Mutations in Igalpha (CD79a) result in a complete block in B-cell development. J Clin Invest 104, 1115-1121 (1999).

12. Szremska, A. P., Kenner, L., Weisz, E., Ott, R. G., et al. JunB inhibits proliferation and transformation in B-lymphoid cells. Blood 102, 4159-4165 (2003).

13. Hartenstein, B., Teurich, S., Hess, J., Schenkel, J., et al. Th2 cell-specific cytokine expression and allergen-induced airway inflammation depend on JunB. The EMBO journal 21, 6321-6329 (2002).

14. Cook, A. D., Braine, E. L., Campbell, I. K. & Hamilton, J. A. Differing roles for urokinase and tissue-type plasminogen activator in collagen-induced arthritis. Am J Pathol 160, 917-926 (2002).

15. Akerblad, P., Rosberg, M., Leanderson, T. & Sigvardsson, M. The B29 (immunoglobulin beta-chain) gene is a genetic target for early B-cell factor. Mol Cell Biol 19, 392-401 (1999).

16. Meyer, K. B., Teh, Y. -M. & Neuberger, M. S. The lgk 3'-enhancer triggers gene expression in early B lymphocytes but its activity in enhanced on B cell activation. International immunology 8, 1561-1568 (1996).

17. Palacios, E. H. & Weiss, A. Function of the Src-family kinases, Lck and Fyn, in T-cell development and activation. Oncogene 23, 7990-8000 (2004).

18. Jain, J., Nalefski, E. A., McCaffrey, P. G., Johnson, R. S., et al. Normal peripheral T-cell function in c-Fos-deficient mice. Mol Cell Biol 14, 1566-1574 (1994).

19. Maynard, C. L. & Weaver, C. T. Diversity in the contribution of interleukin-10 to T-cell-mediated immune regulation. Immunol Rev 226, 219-233 (2008).
